# Whole-genome sequencing of tetraploid potato varieties reveals different strategies for drought tolerance

**DOI:** 10.1038/s41598-024-55669-3

**Published:** 2024-03-05

**Authors:** Florian Schilling, Christina Schumacher, Karin Köhl, Heike Sprenger, Joachim Kopka, Rolf Peters, Manuela Haas, Ellen Zuther, Renate Horn

**Affiliations:** 1 Department of Plant Genetics, Institute of Biological Sciences, University of Rostock, Albert-Einstein-Str. 3, 18059 Rostock, Germany; 2 Max Planck Institute of Molecular Plant Physiology, Am Mühlenberg 1, 14476 Potsdam, Germany; 3 Landwirtschaftskammer Niedersachsen, Dethlingen 14, 29633 Munster, Germany; 4 Present Address: Department of Food Safety, German Federal Institute for Risk Assessment, Max-Dohrn-Straße 8-10, 10589 Berlin, Germany; 5 Present Address: PotatoConsult UG, Hiddinger Straße 33, 27374 Visselhövede, Germany; 6 Present Address: Ministry of Agriculture, Environment and Climate Protection, Henning-Von-Tresckow-Straße 2-13, 14467 Potsdam, Germany; 7 Present Address: Center of Artificial Intelligence in Public Health Research, Robert Koch Institute, Nordufer 20, 13353 Berlin, Germany

**Keywords:** Genetics, Molecular biology, Plant sciences

## Abstract

Climate changes leading to increasingly longer seasonal drought periods in large parts of the world increase the necessity for breeding drought-tolerant crops. Cultivated potato (*Solanum tuberosum*), the third most important vegetable crop worldwide, is regarded as drought-sensitive due to its shallow root architecture. Two German tetraploid potato cultivars differing in drought tolerance and their F1-progeny were evaluated under various drought scenarios. Bulked segregant analyses were combined with whole-genome sequencing (BSA-Seq) using contrasting bulks of drought-tolerant and drought-sensitive F1-clones. Applying QTLseqr, 15 QTLs comprising 588,983 single nucleotide polymorphisms (SNPs) in 2325 genes associated with drought stress tolerance were identified. SeqSNP analyses in an association panel of 34 mostly starch potato varieties using 1–8 SNPs for each of 188 selected genes narrowed the number of candidate genes down to 10. In addition, ent-kaurene synthase B was the only gene present under QTL 10. Eight of the identified genes (*StABP1*, *StBRI1, StKS, StLEA, StPKSP1*, *StPKSP2*, *StYAB5*, and *StZOG1*) address plant development, the other three genes (*StFATA, StHGD and StSYP*) contribute to plant protection under drought stress. Allelic variation in these genes might be explored in future breeding for drought-tolerant potato varieties.

## Introduction

Climate change is likely to increase the frequency and duration of drought events in many regions, especially affecting drought-sensitive crops^1^. The complexity of the drought response and the coordinated optimized processes involved require the application of whole-genome approaches for breeding towards drought tolerance. Furthermore, breeding for climate resilience based on field tests is labour- and time-intensive; therefore, genomic and bioinformatic approaches are essential to facilitate faster and more efficient breeding processes to reduce costs and duration to a minimum^2^.

Potato with a worldwide production of an estimated 359 million tons in 2020 is the third most important nongrain crop and the sixth most important crop worldwide^3,4^. Potatoes are mainly grown in Europe, America and Asia, with more than half of the cultivated areas located in developing countries^5–7^. In addition to serving as an important crop for human nutrition, potato tubers are also important as animal feed and raw material in the starch industry^8,9^. The yield and quality of potato tubers depend heavily on the environmental conditions during cultivation as potatoes are prone to drought stress due to their shallow root architecture^10,11^. However, differences in the root development of potato cultivars could be linked to drought tolerance^12,13^. Choice of irrigation management in growing potatoes plays a major role in yield and tuber quality^14,15^. Considering the predicted climate changes yield losses for potato are expected to range between 18 and 32%^16,17^. Since the late 1990s, northern China, which produces 45% of Chinese potatoes and over 10% of the world’s potatoes, suffered severe periods of drought^6^. In potato, traits such as leaf area expansion, leaf senescence, partitioning of assimilates, tuber initiation and tuber growth are seriously affected by limited water supply^6,18–21^. The cascading effects of drought stress decrease protein and phenolic content of potato tubers, which reduces the benefits of potato for human nutrition^22^. Coping with drought response demands understanding complex networks of interacting genes in plants^23,24^. Profiling the transcriptome showed that in potato a number of different transcription factors, protein kinases, and genes regulating ABA biosynthesis and wax synthesis are involved in the drought stress response^25,26^. Association studies identified allelic variations relevant for drought tolerance in genes of ethylene biosynthesis (*ACS3*), ethylene response (*ERF*), ABA signalling pathway (*PP2C*), DNA repair (*PARG*) and reactive oxygen detoxification (*ALDH*)^27^. Furthermore, QTL analyses in tetraploid potato identified regions of drought tolerance that overlapped with QTLs relevant to tuber starch content and yield^28^.

Bulked segregant analyses (BSAs) have been successfully used to identify molecular markers in segregating F2-populations^29–32^, but are now also applied to whole-genome sequences (BSA-Seq). In potato, this approach was successfully used to identify the nematode resistance gene *H2*^33^ and genes involved in the formation of steroidal glycoalkaloids^34^. Recently, BSA-Seq allowed us to address SNP variations in candidate genes located under QTLs for drought tolerance in the cross of two German potato varieties Albatros and Ramses^28^. BSA-Seq or pooled whole genome sequencing (WGS) has also been utilized to identify agronomically important loci in rice^35^, peach^36^, and melon^37^.

In this study, drought tolerance in tetraploid potato cultivars was approached by using BSA-Seq data for QTL detection and combined with SeqSNP analyses for association studies to narrow down the candidate genes. Ten candidate genes were identified as key players in developmental processes and protection of potato plants under drought conditions. An eleventh gene was the only one present under QTL 10. Allelic variants in the eleven key candidate genes now provide starting points to reveal the mechanisms behind drought tolerance.

## Result

### Evaluation of drought tolerance in cultivated potato varieties and the F1-population

**Figure 1:**
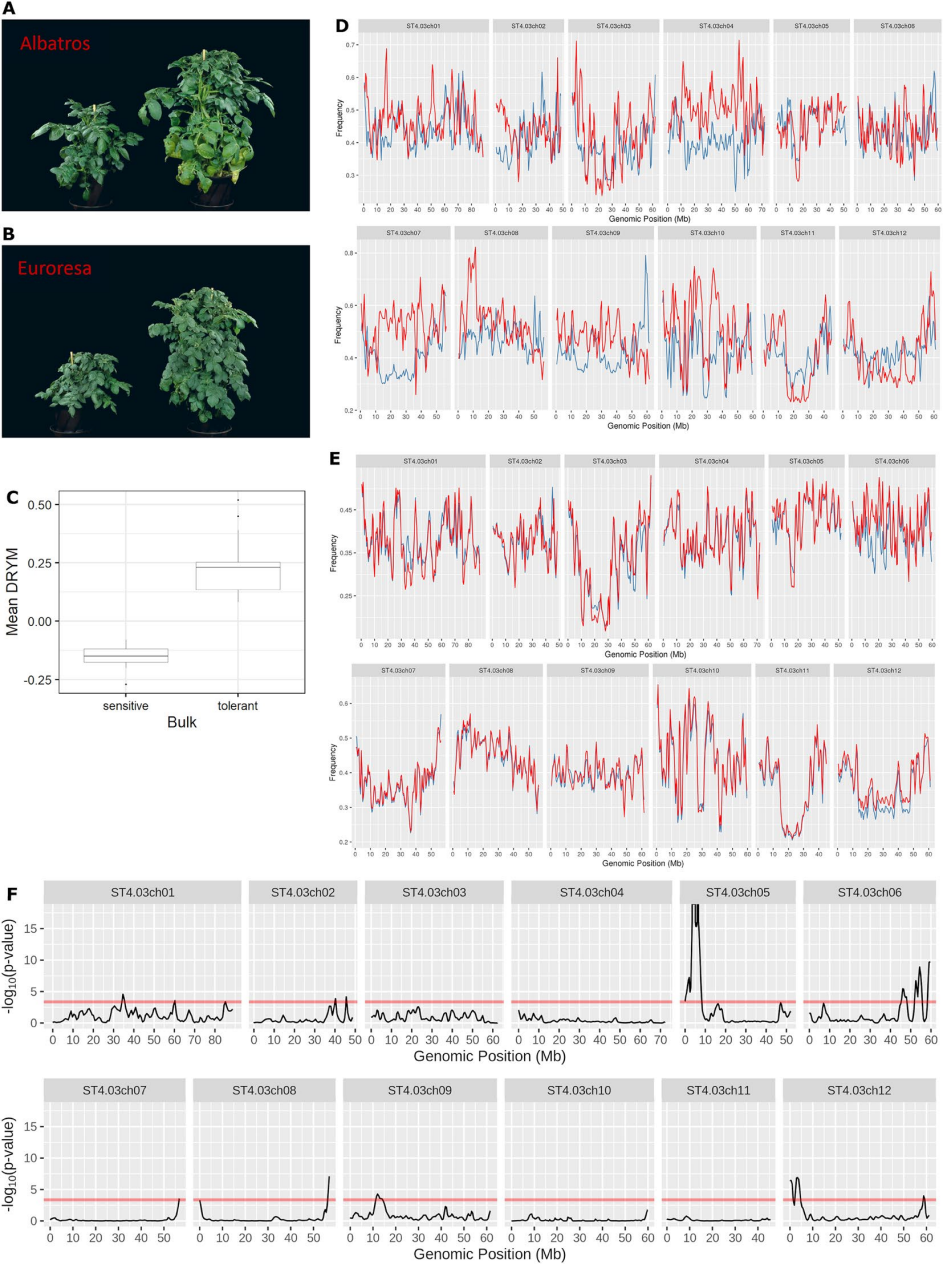
Drought-tolerant and drought-sensitive bulks selected from the F1-progeny of a cross between the varieties Euroresa and Albatros used for whole-genome sequencing and QTL analyses. (**A**) Potato plants of the variety Albatros under drought stress (left) and under well-watered conditions (right). (**B**) Potato plants of the variety Euroresa under drought stress (left) and under well-watered conditions (right). (**C**) Box plot of the mean DRYM values of the F1-clones forming the drought-sensitive bulk and drought-tolerant bulk (significant difference at a p-value of 1.02e−12). (**D**) Allele frequency comparison between all SNPs from the parent cultivars Euroresa (blue) and Albatros (red). The graphs were created in R using the package ggplot2. The x-axis shows the genomic position in Mbp for every chromosome. The y-axis shows the mean allele frequency. The R package WindowScanR was used to calculate frequency means for local windows of the size of 1e^6^ bp. (**E**) Allele frequency comparison between all SNPs from the HROEXASEN (blue) and HROEXATOL (red) bulks. The graphs were created in R using the package ggpot2. The x-axis shows the genomic position in Mbp for every chromosome. The y-axis shows the mean allele frequency. The R package WindowScanR was used to calculate frequency means for local windows of the size of 1e^6^ bp. (**F**) Estimation of differences between the allele frequencies from the drought-tolerant and drought-sensitive bulks by using the G′-value. A threshold was set at p-value = 0.01. The differences in allele frequencies and the G′-values were calculated by using the R package QTLseqr.

This study is based on the whole-genome sequencing of segregating bulks of the F1-progeny derived from a cross Euroresa × Albatros (E × A). Significant differences in drought stress tolerance between the drought-sensitive and drought-tolerant bulk (p-value = 1.02e−12) were detected using the Welch two-sample t-test (Fig. 1A–C). The DRYM values for the drought-sensitive bulk ranged from − 0.27 to − 0.08, with a mean DRYM value of − 0.15, whereas the DRYM values for the drought-tolerant bulk ranged from 0.08 to 0.52, with a mean DRYM value of 0.23.

### Whole-genome sequencing and SNP calling in cultivated potato

Next-generation sequencing (2 × 150 bp paired-end) was performed with 120 × genome coverage to address the genomic constitution of cultivated potatoes, which are autotetraploid (2n = 4x = 48) and highly heterozygous. Two parental potato cultivars, Euroresa and Albatros, and the contrasting bulks were sequenced resulting in 35 GB of data per sample. SNP calling was performed against the assembled diploid potato genome DM v4.03^38^. The parental cultivars Albatros (HROALB) and Euroresa (HROEUR) showed a total of 21,190,336 and 25,098,177 variants, with 18,502,085 and 19,560,602 being actual SNPs, respectively (Supplementary Table S1). The drought-sensitive bulk (HROEXASEN) and the drought-tolerant bulk (HROEXATOL) yielded 24,272,018 and 24,057,945 variants with 21,394,166 and 21,200,308 SNPs, respectively. The difference between variants and SNPs consisted of INDELs. The number of total INDELs ranged from 11.2 to 12.7% of the total variants. To verify the efficiency of SNP calling our SNP data were compared to the set of 69,011 high confidence SNPs reported by Hamilton et al. 2011 and the SolCap array with 8303 SNPs^39,40^. Most of the reported SNPs were also identified in our work using Albatros and Euroresa (83.2% and 83.5%, respectively) confirming the high quality of our SNP calling.

### Comparison of SNP allele frequencies and drought tolerance associated QTLs

Due to the high number of SNPs per cultivar ranging from 21.2 to 25 million, windows summing SNP frequencies had to be used for visualization purposes and to obtain a basic overview about the differences in SNP allele frequencies (Fig. 1D,E). Regarding the bulks, the filtering steps reduced the approximately 20 million SNPs to approximately 6 million SNPs that were used for QTL mapping by QTLseqr. Most of these SNPs represent differences towards the diploid potato genome assembly that are present in both bulks and are not relevant for the investigations. Only 588,983 SNPs correspond to differences between the drought-tolerant or the drought-sensitive bulk, which shows that most SNPs originate from differences between the diploid reference genome and the tetraploid genome of the potato cultivars. Comparing the drought-tolerant and drought-sensitive bulks from E × A 15 QTLs were detected: three QTLs on chromosomes 1, 6 and 12, two QTLs on chromosome 2 and one QTL on chromosomes 5, 7, 8 and 9, respectively (Fig. 1F).

Under all 15 QTLs together, a total of 2325 annotated genes were located showing 589,463 SNPs towards the reference genome and only 23,722 unique SNPs for either the drought-sensitive or the drought-tolerant bulk (Table 1). QTL 3 on chromosome 1 is the smallest with only 41,187 bp, but also the QTL showing the highest gene density with 292 genes per Mbp. The longest QTL is QTL 6 on chromosome 5 with a length of 8,263,689 bp and 753 genes. QTL 10 on chromosome 7 spanning 63,682 bp covers only a single gene encoding an ent-kaurene synthase 9. All QTLs together spanned a total of 27.2 Mbp (3.24%) of the potato genome.

**Table 1:** Overview of all identified drought stress associated QTLs in Euroresa × Albatros, showing the QTL ID, the chromosome on which the QTL is located, the region of the QTL on the chromosome, the number of genes under the QTL and the number of SNPs for each QTL.

QTL	Chrom	Region [bp]	Area [bp]	Total genes	Gene density [genes/Mbp]	Total SNPs
1	Chr1	34,124,274–35,482,929	1,358,655	33	25	31,129
2	Chr1	60,085,436–60,330,764	245,328	14	58	6246
3	Chr1	85,175,189–85,216,376	41,187	12	292	999
4	Chr2	39,999,989–40,404,248	404,259	45	112	10,045
5	Chr2	45,414,396–45,796,712	382,316	47	123	3040
6	Chr5	88–8,263,777	8,263,689	753	92	192,743
7	Chr6	45,262,468–48,153,426	2,890,958	232	81	61,076
8	Chr6	51,855,603–55,730,666	3,875,063	414	107	61,111
9	Chr6	58,242,043–59,532,090	1,290,047	150	117	24,262
10	Chr7	56,689,152–56,752,834	63,682	1	16	873
11	Chr8	56,234,261–56,933,939	699,678	70	101	12,778
12	Chr9	11,242,995–14,240,453	2,997,458	99	34	76,843
13	Chr12	19,226–1,446,194	1,426,968	150	106	30,868
14	Chr12	2,120,460–4,718,278	2,597,818	244	94	63,849
15	Chr12	58,579,071–59,261,529	682,458	61	90	13,601
Σ			27,219,564	2325	96.5	589,463

Genomic locations are given according to reference version DM v4.03.

**Figure 2:**
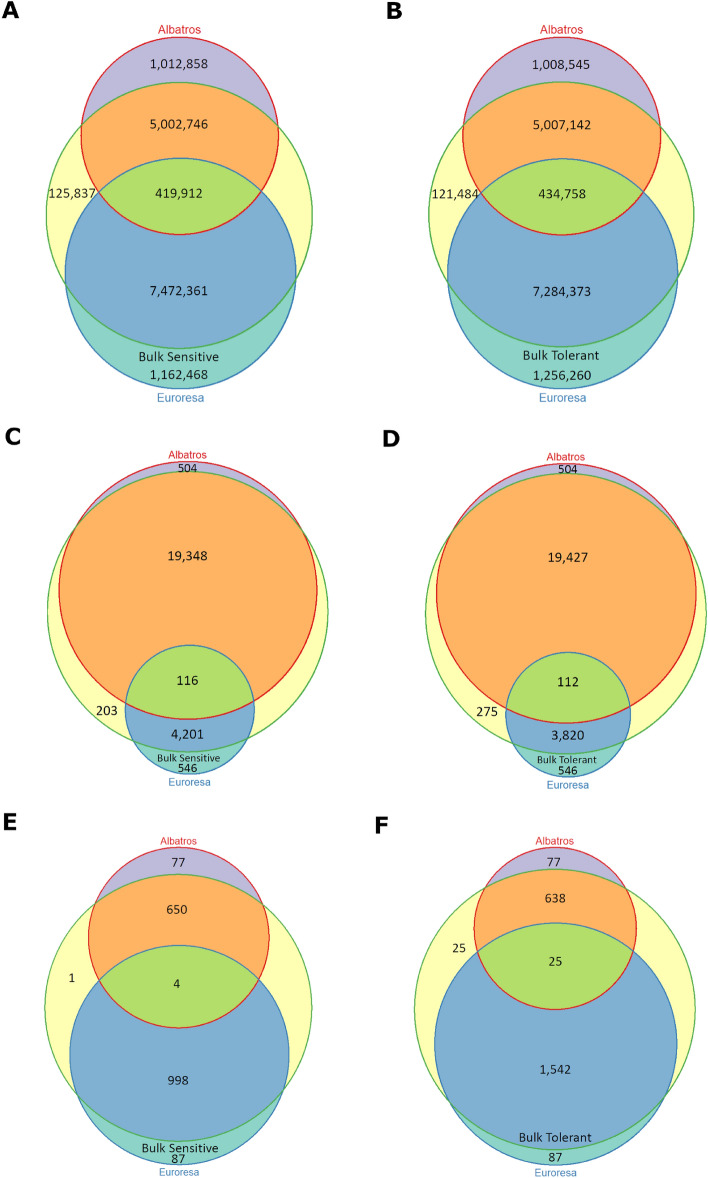
Comparison of filtered SNPs over the whole genome and in selected QTL regions visualized in Venn diagrams by using the R package Vennerable. The red circle represents Albatros, the blue circle Euroresa and the green circle either the drought-sensitive (**A**) or tolerant (**B**) bulk. A SNP that is present in Euroresa and one of the bulks is represented by the blue area, a SNP present in Albatros and one of the bulks by the orange area. SNPs present in both parents and the bulk are represented by the green area. (**C**) and (**D**) Comparison of filtered SNPs in the genome region covered by QTL 1. The majority of SNPs under QTL 1 are contributed by Albatros. (**E**,**F**), Comparison of filtered SNPs in the genome region covered by QTL 5. The majority of SNPs under QTL 5 are contributed by Euroresa.

Comparing all filtered SNPs present in the parents and in one of either bulks, showed that regarding the whole genome the cultivar Euroresa adds more SNPs to the bulks than the cultivar Albatros (Fig. 2A,B). For the drought-tolerant bulk 38.97% of the SNPs originated from Albatros and 56.70% came from Euroresa. Only 3.38% of SNPs were present in both parents and the drought-tolerant bulk.

**Figure 3:**
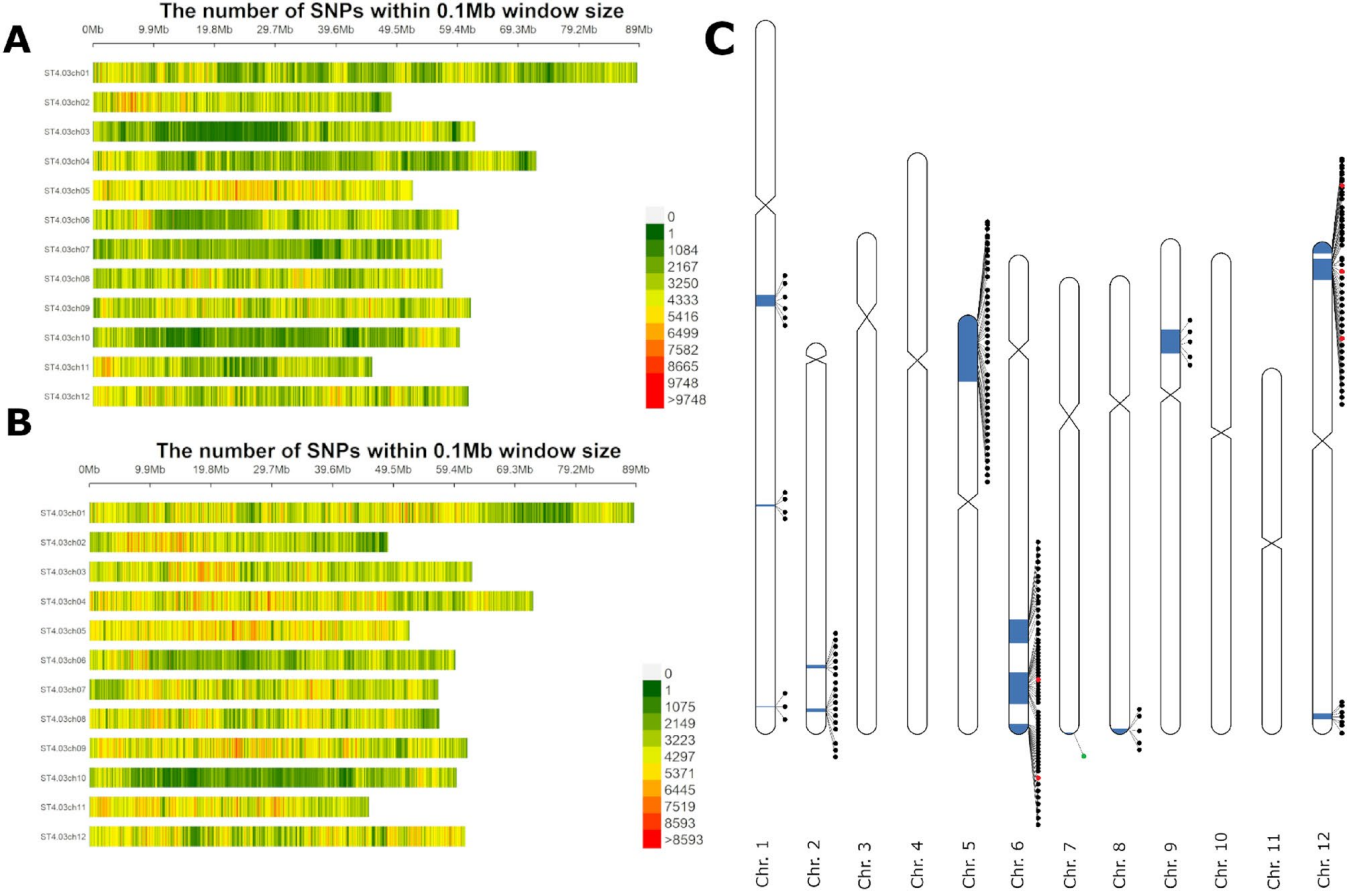
Distribution of SNPs and drought tolerance (blue) QTL in the cross E × A with locations of 188 candidate genes, which were further used for SeqSNP analyses in association studies. (**A**) SNP distribution over the whole genome of Albatros. (**B**) SNP distribution over the whole genome of Euroresa. (**C**) Distribution of genes used for SeqSNP analyses (black dots). Genes tagged by SNPs significantly associated with drought tolerance by the exact Fisher test are shown in red. Ent-kaurene synthase B, the only gene under QTL 10, is marked with a green dot. Locations obtained from the DM v4.03 assembly were used in this figure.

For the drought-sensitive bulk, the percentages were very similar: 38.42% of the SNPs were present in Albatros and the drought-sensitive bulk, 57.39% of the SNPs were present in Euroresa and the drought-sensitive bulk. Only 3.22% were present in all three. For the individual QTL the situation looked different. The two QTLs with SNPs, the most off balance towards one parent, were QTL 1 and QTL 5. For QTL 1, 81.06% of SNPs present in the drought-sensitive bulk and 82.20% of the SNPs in the drought-tolerant bulk originated from Albatros (Fig. 2C,D). On the other hand, only 17.60% of the SNPs for the drought-sensitive bulk and 16.16% of the SNPs for the drought-tolerant bulk came from Euroresa. For QTL 5, 60.38% of the SNPs in the drought-sensitive bulk and 69.15% in the drought-tolerant bulk came from Euroresa, whereas only 39.32% of the SNPs present in the drought-sensitive bulk and 28.61% in the drought-tolerant bulk originated from Albatros (Fig. 2E,F). Differences in the SNP distribution over the whole genome are also visible between Albatros and Euroresa (Fig. 3A,B).

### Association studies using SNP variations detected by SeqSNP analyses

For verification, SNPs tagging candidate genes underlying the 15 identified QTL regions were selected in two rounds. In the first round, only SNPs in genes for drought tolerance described in the literature (coding sequence plus 2000 bp 5′-UTR and 500 bp 3′-UTR) were considered. SNPs had to be exclusively present in either the drought-tolerant or the drought-sensitive bulk. To obtain a higher coverage of candidate genes that could be analyzed, a maximum of 2–3 SNPs per gene were selected for SeqSNP analyses. This resulted in 449 SNPs corresponding to 206 genes. For the second round, only SNPs leading to missense or nonsense mutations between the bulks were selected, but all 2325 annotated genes under the 15 QTL were included, which resulted in an additional 585 SNPs for another 120 genes. In total, 1034 SNPs representing 324 candidate genes were obtained by this combined strategy to conduct further association studies for drought tolerance in a panel of 34 mostly German starch potato cultivars. The 324 candidate genes for drought tolerance are distributed under all 15 QTL regions. However, heterozygosity and complexity of the tetraploid potato genome only allowed an oligonucleotide design for 410 SNPs (39.6%, Supplementary Table S2) to go into the SeqSNP analysis, which represented 188 candidate genes (Fig. 3C).

Association studies using the SeqSNP data from the potato association panel revealed seven SNPs to be significantly associated with drought tolerance corresponding to six genes (Table 2). Two of the genes (acyl-ACP thioesterase, Soltu.DM.06G033680.1 and ER auxin binding protein 1, Soltu.DM.06G034890.1) were located in the distal region of chromosome 6 under QTL 9 and the other four genes were positioned under QTLs 13 and 14 at the proximal end of chromosome 12 (Fig. 3C). These genes encode homogentisate 1,2-dioxygenase (Soltu.DM.12G029920.1), a plant specific transcription factor with a YABBY domain (Soltu.DM.12G026680.1), and two proteins belonging to kinase superfamily proteins (Soltu.DM.12G026420.1 and Soltu.DM.12G026450.1). In addition, a single gene encoding ent-kaurene synthase B (Soltu.DM.07G028660.1) was present under QTL 10.

**Table 2:** Association with drought tolerance in the potato association panel and classification of candidate SNPs.

SNP ID	Chromosome	Position	Ref	Alt	p-value	Type	Gene ID	Gene name
Soltu.DM.06G033680.1_SNP5	ST4.03ch06	57,938,560 [58,293,229]	C	G	0.0184*	Missense variant	Soltu.DM.06G033680.1	*StFATA*
Soltu.DM.06G034890.1_SNP2	ST4.03ch06	58,819,351 [59,199,152]	T	C	0.0167*	5ʼ-UTR variant	Soltu.DM.06G034890.1	*StABP1*
Soltu.DM.12G026420.1_SNP1	ST4.03ch12	56,378,682 [3,034,965]	C	A	0.0255*	3ʼ-UTR variant	Soltu.DM.12G026420.1	*StPKSP1*
Soltu.DM.12G026450.1_SNP1	ST4.03ch12	56,390,498 [3,046,881]	T	C	0.0134*	3ʼ-UTR variant	Soltu.DM.12G026450.1	*StPKSP2*
Soltu.DM.12G026680.1_SNP2	ST4.03ch12	56,600,071 [2,821,461]	C	T	0.0004***	3ʼ-UTR variant	Soltu.DM.12G026680.1	*StYAB5*
Soltu.DM.12G026680.1_SNP1	ST4.03ch12	56,600,575 [2,820,957]	T	G	0.0039**	3ʼ-UTR variant		
Soltu.DM.12G029920.1_SNP4	ST4.03ch12	59,287,788 [363,947]	C	A	0.0149*	Missense variant	Soltu.DM.12G029920.1	*StHGD*

Polymorphisms (SNP ID) are shown with indication of the exact Fisher test p-value, the chromosome, the position on the chromosome, the nucleotide (Ref) for the reference genome at the position of the SNP and the alternative nucleotide (Alt) for the position of the SNP. Significance levels: p < 0.05*, p < 0.01**, p ≤ 0.001***. The p-values were obtained from the SeqSNP analyses. Gene ID, position, type of mutation and gene name are given for each significantly associated SNP. SNPs were remapped to the genome assembly and annotation v6.1 for *S. tuberosum* Group Phureja DM 1–3 516 R44^38^. Hence, locations and gene IDs are according to the v6.1 genome annotation. The corresponding position in the former v4.3 genome annotation is given in brackets.

Performing Kruskal–Wallis’s analysis instead of the exact Fisher test, four of the genes (Soltu.DM.06G033680.1, Soltu.DM.12G029920.1, Soltu.DM.12G026680.1 and Soltu.DM.12G026450.1) having the highest impact on the phenotypic variance η^2^ were confirmed (Supplementary Table S3), whereas no association was detected for the two other genes Soltu.DM.06G034890.1 and Soltu.DM.12G026420.1. However, four additional SNPs were significantly associated with drought tolerance tagging two additional genes (Soltu.DM.12G026610.1 and (Soltu.DM.12G027240.1) under QTL 14 on chromosome 12 and two genes (Soltu.DM.02G024960.1 and Soltu.DM02G025020.1) on chromosome 2, both located under QTL4.

### Mutations in seven identified candidate genes for drought tolerance in tetraploid potatoes

**Figure 4:**
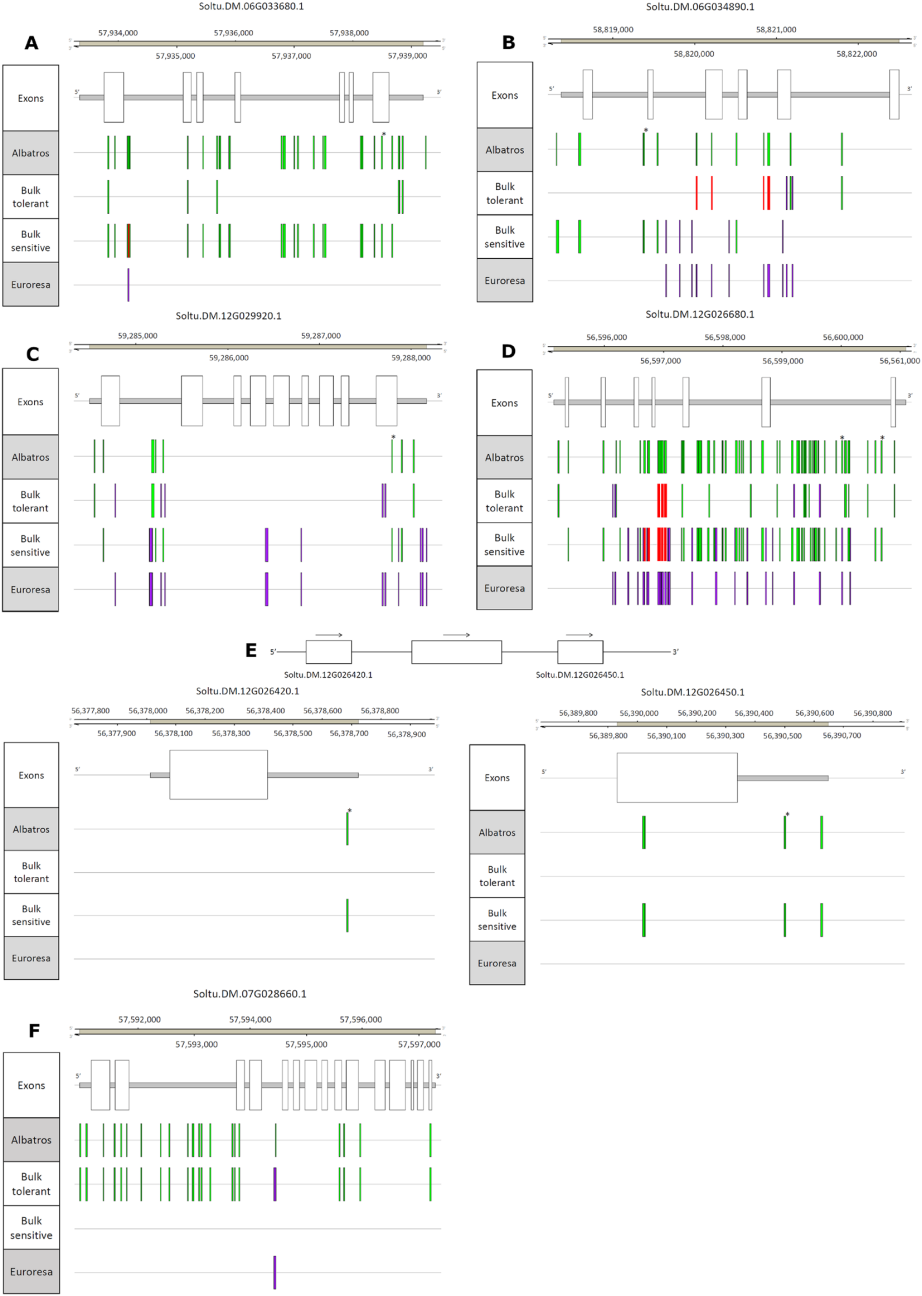
SNP distribution in seven identified genes for drought tolerance in potato. (**A**) Visualization of SNPs located in the gene Soltu.DM.06G033680.1 (*StFATA*) using the R package Gviz. The first track shows the location of *StFATA* (gray) according to the DM v6.1 genome annotation. The following exon track shows the exact location of the exons. The subsequent four tracks give the exact positions of SNPs, insertions and deletions in the parents Albatros and Euroresa, as well as in the drought-tolerant and drought-sensitive bulk. SNPs are shown in different colors depending on their origin: green (Albatros), purple (Euroresa) and red (present in both parents), asterisks mark significantly associated SNPs. (**B**) Visualization of SNPs located in the Soltu.DM.06G034890.1 gene (*StABP1*) using the R package Gviz. Tracks and colors are the same as used in Fig. 4A. (**C**) Visualization of SNPs in the Soltu.DM.12G029920.1 gene (*StHGD*) by using the R package Gviz. Tracks and colors are the same as used in Fig. 4A. (**D**) Visualization of the SNPs located in the Soltu.DM.12G026680.1 gene *(StYAB5*) using the R package Gviz. Tracks and colors are the same as used in Fig. 4A. (**E**) Visualization of SNPs located in the duplicated genes encoding protein kinase superfamily proteins by using the R package Gviz. Soltu.DM.12G026420.1 (left, *StPKSP1*) and Soltu.DM.12G026450.1 (right, *StPKSP2*). Tracks and colors are the same as used in Fig. 4A. (**F**) Visualization of the SNPs located in the Soltu.DM.07G028660.1 gene (*StKS*) by using the R package Gviz. Tracks and colors are the same as used in Fig. 4A.

Targeted genotyping by sequencing identified seven SNPs located in six genes that were significantly associated with drought tolerance via the exact Fisher test (Table 2, Supplementary Table S3). Comparing the whole gene sequences with the diploid reference genome assembly SolTub v6.0 revealed further differences specific for Albatros and Euroresa. The acyl-ACP thioesterase A, Soltu.DM.06G033680.1 (*StFATA*), under QTL 9 on chromosome 6 was tagged by SNP Soltu.DM.06G033680.1_SNP5 at position 57,938,362 bp, which is significantly associated with drought tolerance (p-value = 0.0184). This SNP leads to a missense mutation Asp > Asn in the variety Albatros (Fig. 4A). The acyl-ACP thioesterase at 57,933,541 to 57,938,992 bp (+ strand) has the highest homology to AT4G13050.1 (*FATA2*) encoding an oleoyl-acyl-carrier protein hydrolase (acyl-ACP thioesterase) in *Arabidopsis thaliana.* Twenty-eight SNPs are located within *StFATA*, which apart from two are present in either one of the bulks. Six of the SNPs are located in the coding region, including three missense mutations (Asp > Asn at 57,938,362 bp, Arg > Lys at 57,933,824 bp and Lys > Arg at 57,933,938 bp). The most influential structural changes next to the missense mutations are two deletions at 57,936,818 bp and 57,937,512 bp, deleting ACA and TT, respectively. One insertion at 57,936,803 bp, adds a T in intron 4. In *StFATA*, 27 of 28 SNPs originated from Albatros and only one SNP at 57,934,172 bp compared to the diploid potato genome was present in both parents. Twenty-two SNPs were present in the drought-sensitive bulk, and four SNPs from Albatros were only present in the drought-tolerant bulk. The single SNP occurring in Euroresa at position 57,934,172 contributed a silent mutation in the drought-sensitive bulk. The described two deletions and the one insertion were only present in Albatros and the drought-sensitive bulk.

The gene coding for ER auxin binding protein 1 (*StABP1*), also located under QTL 9, was targeted by Soltu.DM.06G034890.1_SNP2 (p = 0.01669). *StABP1* starts at position 58,822,497 and ends at 58,818,364 on chromosome 6 (+ strand) and is homologous to AT4G02980.1. In total, 23 SNPs were detected in *StABP1*. The significantly associated SNP (p-value = 0.01669) that targeted *StABP1* represents an intron variant at 58,819,351 bp (Fig. 4B). Five other SNPs represent mutations in exon regions of *StABP1*, two resulting in missense mutations. The first missense mutation is located at 58,820,187 bp resulting in a conservative replacement Phe > Tyr. However, the second missense mutation located at 58,821,105 bp represents a radical replacement Thr > Ile. Two insertions at 58,818,289 bp and 58,820,872 bp, the former in the 5’ UTR in Albatros and the drought-sensitive bulk and the latter in intron 4 in Euroresa and the drought-tolerant bulk were located next to the missense mutations. An additional deletion of the sequence ACGCTAGACCCC occurred at 58,818,560 bp. Fifteen of the 23 SNPs originated from Albatros and 14 from Euroresa. Ten SNPs were only present in the drought-tolerant bulk, and 13 were present in the drought-sensitive bulk. Both missense mutations coming from Euroresa are only present in the drought-tolerant bulk, while the missense mutation at 58,820,187 bp originating from Albatros can be found in both contrasting bulks. The remaining SNPs resulted in silent mutations.

The gene encoding homogentisate 1,2-dioxygenase (*StHGD*, Soltu.DM.12G029920.1), on chromosome 12 in QTL 13, is involved in tyrosine breakdown. The homologue in *Arabidopsis* is AT5G54080. *StHGD* was targeted by the significant drought tolerance-associated SNP Soltu.DM.12G029920.1_SNP4 (p-value = 0.01492) at position 59,287,789 in the coding region of the gene (Fig. 4C). This SNP results in a conservative missense mutation Gly > Val present in Albatros and the drought-sensitive bulk. In total, five SNPs are located in the coding region of *StHGD*, and two represent missense mutations. The first tagged the gene, and the second was located at 59,284,638 bp and led to a conservative amino acid change Ser > Thr in Albatros and the drought-sensitive bulk. In addition, one insertion and two deletions were present in *StHGD*. The insertion at 59,287,184 bp and the first deletion at 59,285,139 bp were present in Euroresa and the drought-sensitive bulk. The insertion adds a T and the deletion removes nine nucleotides (TAACTTATC). Both mutations were located in noncoding regions. The second deletion TA within intron 1 at 59,285,167 was present in Albatros and the drought-tolerant bulk. At the *StHGD* locus, 20 SNPs were identified, that fit the criteria of being present in only one of the contrasting bulks. Eight of the 20 SNPs originated from Albatros and 12 from Euroresa. Seven SNPs (five originating from Euroresa, two from Albatros) and one deletion were only present in the drought-tolerant bulk. In the drought-sensitive bulk, 13 SNPs as well as one deletion and one insertion were unique, with eight of the SNPs originating from Euroresa. The 13 SNPs resulted in only two missense mutations as described before, five mutations in the 3’UTR, six mutations in introns and one mutation in the 5’UTR.

The plant-specific transcription factor with a YABBY domain encoded by Soltu.DM.12G026680.1 (*StYAB5*) under QTL 14 (Chr. 12) showed homology to *YAB5* in *Arabidopsis* (AT2G26580.1) and was targeted by two SNPs that were significantly associated with drought tolerance. In total, seventy-one SNPs were present in either one of the bulks in *StYAB5* (Fig. 4D). Six SNPs were located in the coding region, and four of them caused missense mutations. These missense mutations are located at 56,595,979 bp (Met > Arg), 56,597,336 bp (Arg > Met), 56,598,724 bp (Asn > Asp) and 56,600,893 bp (Thr > Ala). Three of the mutations represent radical replacements, while Asn > Asp can be regarded as a conservative replacement. In addition, a total of 13 deletions and eleven insertions were located in *StYAB5*. The longest deletion removed AAACTCTAGAG from the sequence at position 56,596,989 bp. The longest insertion, on the other hand, was located at 56,599,076 bp and added eight thymidines to the sequence. Of all 71 SNPs, 52 originated from Albatros and 19 from Euroresa. The drought-tolerant bulk was characterized by 19 SNPs and the drought-sensitive bulk by 52. The two significant drought tolerance-associated SNPs Soltu.DM.12G026680.1_SNP1 (at position 56,600,575 bp, p = 0.003859) and Soltu.DM.12G026680.1_SNP2 (at position 56,600,071 bp, p = 0.00041) both represent point mutations in intron 6 of *StYAB5*. Both SNPs were only present in Albatros and the drought-sensitive bulk.

The genes Soltu.DM.12G026420.1 and Soltu.DM.12G026450.1 encoding protein kinase superfamily proteins (*StPKSP1* and *StPKSP2*) are arranged in tandem array on chromosome 12 (QTL 14), with homology to AT4G31170. Both genes were targeted by one significantly associated SNP each, but the SNPs differed between the two genes (Fig. 4E). All five SNPs, present in the two duplicated genes originated from Albatros and were exclusively present in the drought-sensitive bulk: one in Soltu.DM.12G026420.1 and four in Soltu.DM.12G026450.1. Two SNPs were located in the coding region of Soltu.DM.12G026450.1, but only one resulted in a missense mutation with a radical amino acid exchange Pro > Ser at position 56,390,021 bp. Both significantly associated SNPs were located in the 3’-UTR-region of the genes: Soltu.DM.12G026420.1_SNP1 (p-value = 0.01336) at position 56,378,682 bp and Soltu.DM.12G026450.1_SNP1 (p-value = 0.02551) at 56,390,498 bp.

Furthermore, Soltu.DM.07G028660.1 (*StKS*), encoding ent-kaurene synthase B, was also defined as a candidate gene, because it was the only gene present in the narrow QTL 10 on chromosome 7. The homologue in *Arabidopsis* is AT1G79460. Ent-kaurene synthase B is located at 57,590,958 to 57,597,303 bp (+ strand). In total, 19 SNPs were detected in Soltu.DM.07G028660.1, five of which were in the coding region with a single radical missense mutation due to a C > T exchange at position 57,591,796, changing Ala > Thr (Fig. 4F). In addition, there were two deletions located in noncoding regions in ent-kaurene synthase B. One was present in Albatros and the drought-tolerant bulk at 57,591,066 bp in the 5’UTR, deleting AAGA, and the other deletion was present in Euroresa and the drought-tolerant bulk at 57,594,426 bp in the 3’UTR, deleting a single guanidine. All 19 SNPs from Albatros were only present in the drought-tolerant bulk.

For the four additional genes identified by the Kruskal–Wallis test, the significantly with drought tolerance associated SNPs as well as the comparison of the gene sequences of Albatros and Euroresa are given in Supplementary Table S3 and Supplementary Fig. S1.

## Discussion

Not all genetic variation present in the tetraploid potato genome can be displayed by the double monoploid reference genome^38^. Twenty to 25 million SNPs were called in comparison to the diploid reference genome, while the number of filtered SNPs present in one of the bulks comprised only a total of 588,983. A tetraploid reference genome would considerably decrease the amount of data that has to be analysed without losing relevant information. The combination of BSA-Seq for QTL analyses with SeqSNP data for association studies proved to be very efficient in the identification of eleven candidate genes significantly associated with drought tolerance in potato (Supplementary Table S4). However, these genes might not be the only genes relevant for the QTL, as neighbouring genes could also be involved depending on the linkage disequilibrium. Even though Albatros represents the drought-tolerant parent, the results clearly show that Euroresa might also contribute alleles relevant for drought tolerance and vice versa. The identified QTLs for drought tolerance in this study partially show overlaps with QTLs for drought response and drought tolerance in previous publications (Supplementary Table S5)^27,41,42^.

Recently, SeqSNP and KASP analyses proved to be very efficient and cost effective in genotyping South African potato cultivars^43^. In our study, ten genes were tagged by SeqSNP analyses as candidates for drought tolerance, and an eleventh gene was identified as the only gene under QTL 10 on Chr. 7. The acyl-ACP thioesterase A (*StFATA*, Soltu.DM.06G033680.1) was tagged by a SNP representing drought-sensitive allele(s) as the SNP, even though coming from Albatros, was exclusively present in the drought-sensitive bulk. However, four other SNPs specific for Albatros were only present in the drought-tolerant bulk indicating additional drought-tolerant allele(s) in Albatros. The drought-sensitive allele(s) carried two missense mutations Lys > Arg and Asp > Asn. Two classes of acyl-ACP thioesterases, FATA and FATB, can be distinguished^44^. FATA thioesterases prefer unsaturated fatty acids such as 18:1 in vitro. In tomato, a 1.7 times higher *FATA* expression level under drought stress resulted in an increase in phospholipids as well as remodeling of phospholipids leading to higher membrane stability and better protection of seeds from desiccation^45,46^. FATB thioesterase (Arabidopsis homolog AT1G08510) is part of cuticular wax biosynthesis^47^. In *A. thaliana*, wax synthesis increased up to 75% under water deficit^48,49^. Poplars overexpressing Acyl-ACP thioesterase B showed better stress tolerance^50^.

For ER auxin binding protein 1 (*StABP1*, Soltu.DM.06G034890.1), SNPs from both parents were present in both bulks. ABP1 functions as an auxin receptor, that is involved in signal transduction under abiotic stress^51^. Overexpression of ABP1 leads to increased K + intake into guard cells and stomatal closure^52^.

For ent-kaurene synthase B (*StKS*, Soltu.DM.07G028660.1), all SNPs from Albatros were present in the drought-tolerant bulk indicating a major contribution to drought tolerance from Albatros. Euroresa only contributed a single deletion in an intron region. As part of gibberellic acid (GA) biosynthesis, ent-kaurene synthase directly influences the GA content in plants^53^. Plants with decreased GA concentrations showed better drought tolerance by osmoregulation, inhibited canopy growth, accelerated stomatal closure, reduced xylem expansion and increased root-to-shoot ratio^54^. In rice, ent-kaurene synthase was downregulated under drought stress^55^. In *Stevia rebaudiana*, inhibition of ent-kaurene synthase with chlorcholine chloride increased drought tolerance^56^.

Homogentisate 1,2-dioxygenase (*StHGD*, Soltu.DM.12G029920.1) is involved in tyrosine and homogentisate breakdown. SNP distribution in the bulks indicated that both parents carried drought-sensitive and drought-tolerant allele(s). Homogentisate can be either oxidized by HGD or used as a precursor for tocopherols^57^. Potato and sweet potato mutants with increased tocopherol content have shown higher tolerance to drought stress^58,59^. Mutations inhibiting HGD from catabolizing homogentisate could lead to higher tocopherol production and thus higher drought tolerance. In a cross-species meta-analysis of progressive drought, HGD showed upregulation under drought stress^60^.

YABBY transcription factors modulate morphogenesis, development and stress responses^61^. The small, plant-specific gene family contains five members^62,63^. *StYAB5* (StYAB5, Soltu.DM.12G026680.1) was targeted by two SNPs showing significant associations with drought tolerance. Both parental varieties carry drought-sensitive and drought-tolerant allele(s). Arabidopsis *yab5-1*, a TILLING mutant, has smaller leaves than wild-type Columbia erecta^64,65^. *YABBY* genes carry two conserved domains: a zinc finger domain in the N-terminal region and a YABBY domain in the C-terminal region^66^. *YABBY* genes are part of regulatory processes in salt and drought stress resistance^67–70^. In *Brassica napus*, cis-regulatory elements involved in abiotic stress responses such as MYB-binding sites, response elements for abscisic acid (ABRE), gibberellin P-Box and methyl jasmonate motives (CGTCA- and TGACG), were identified in *YABBY* genes^71^.

Soltu.DM.12G026420.1 and Soltu.DM.12G026450.1 (*StPKSP1* and *StPKSP2*) coding for protein kinase superfamily proteins show the highest homology to the RAF-like MAPKKK 28*.* Both genes were tagged by SNPs indicating a drought-sensitive allele, as the SNPs only occurred in the drought-sensitive bulk. In cotton, virus-induced gene silencing of RAF-like MAPKKK enhanced tolerance to drought and salt^72,73^. RAF-like MAPKKK 28 plays a role in embryogenesis and auxin polar transport. Inactivation in *A. thaliana* resulted in confused localization of the auxin transporters PIN1 and PIN7^74^.

Soltu.DM.12G026610.1 (*StBRI*, Serine/threonine protein kinase BRI1) and Soltu.DM.12G027240.1 (*StZOG1*, Zeatin-O-glucosyltransferase) are located under QTL 14 on chromosome 12 as *StPKSP1*, *StPKSP2* and *StYAB5*. Brassinosteroid Insensitive 1 (BRI1) as receptor represents the starting point of the brassinosteroid signaling pathway, which might be involved in generating stress memory^75^. Zeatin-O-glucosyltransferase plays a role in cytokinin homeostasis by reversibly inactivating cytokinin by glycosylation^76^.

Soltu.DM.02G024960.1 (*StSYP*, Syntaxin) and Soltu.DM02G025020.1 (*STLEA*, Late Embryogenesis Abundant) are both candidate genes for drought response located under QTL4 on chromosome 2. Syntaxin is involved in root meristem growth via regulating the reactive oxygen species (ROS) homeostasis^77^. LEA proteins play a well-known role in cell stabilization under drought conditions^78^.

In conclusion, eight of the identified genes (*StABP1*, *StKS, StLEA, StPKSP1*, *StPKSP2*, *StBRI1, StYAB5*, and *StZOG1*) address plant growth, which has to be well balanced under drought conditions. The other three genes, *StFATA, StSYP* and *StHGD****,*** contribute to protection under abiotic stress, addressing transpiration by playing a role in fat and wax metabolism, ROS homeostasis and protection against intense light by the production of tocopherols. SNP distribution in the contrasting bulks showed that for most of the eleven genes, both varieties contributed SNPs to both bulks indicating that both parents carried drought-sensitive as well as drought-tolerant allele(s) for the genes. For two genes, *StFATA* and *StKS,* only SNPs originating from Albatros seemed to contribute to drought tolerance. On the other hand, for *StPKSP1* and *StPKSP2*, Albatros contributed the drought-sensitive allele(s). For *StSYP*, only SNPs from Euroresa contributed to the drought-tolerant bulk. Potato severely suffers under water deficit^79^. Exploiting allelic variation in the eleven identified genes might confer improved drought tolerance to potato.

## Methods

### Plant material and drought stress treatments

Based on the former ranking of 34 potato varieties by the drought tolerance index DRYM, Albatros represents a drought-tolerant and Euroresa a drought-sensitive variety (Supplementary Table S6)^27^. Both selected parents, Albatros (Norika, Groß Lüsewitz, Germany) and Euroresa (Europlant Pflanzenzucht, Lüneburg, Germany) represent German starch potato varieties (2n = 4x = 48, *Solanum tuberosum* L.) cultivated for industrial purposes with high starch contents of approximately 22% and 21%, respectively. Euroresa is medium late to late from maturity (140–160 days) and Albatros medium early (120–140 days). An F1-progeny was produced by crossing Euroresa (E) with Albatros (A) and maintained by the Max-Planck Institute of Molecular Plant Physiology, Potsdam, Germany. DNA was extracted from leaves according to the method of Doyle and Doyle^80^.

Three drought trials (big-bag trial B2, Golm poly tunnel; field trial F2, Groß Lüsewitz, rain-out shelter, and pot trial P3, JKI Groß Lüsewitz, rain-out shelter) were performed for the two German parental potato cultivars Euroresa and Albatros and the segregating F1 progeny (E × A, 100 clones). Experimental trials were performed under naturally fluctuating climate conditions in 2014 and early drought stress treatments were applied as previously described in detail^81,82^.

In the big-bag trial (B2), drought stress began at the five-leaf stage and was carried on until maturity (> BBCH 90). Drip-irrigation was used to water the plants and drought stress was achieved by prolonging the time interval between two irrigations to limit the irrigation volume to 50% of the water given to the optimally watered plants^82,83^. In the field trials (F2), potatoes were only once irrigated at the start of the experiment to enable the emergence of the plants. For the drought stress simulation, the plants growing under a shelter did not receive any further irrigation until the harvest. The control plants were watered in addition to the normal precipitation to guarantee optimal water conditions. In the pot trials (P3), drought treatment started at the three-leaf stage. The plants endured an ongoing change between drought periods and irrigation. Ten days into the drought phase plants were irrigated. The quantity was equivalent to three times the volume evaporated by potato plants when half of them showed turgor loss. In the control block, the weight loss due to evaporation was replaced daily to maintain a water capacity of 50%. Details of the irrigation, design and micrometeorological conditions were described previously^83^. Raw data are available from E!dal^84^. Subsequent data analyses were performed in SAS 9.4 (SAS Institute). Drought tolerance was assessed based on the tuber starch yield (SY), which is the product of tuber mass and starch content. To facilitate the comparison of experiments, the relative SY of each genotype (G) was normalised as the deviation of the relative starch yield from the median of the experiment (E) as given in Eq. (1).

1 $$RelS{y}_{GXEi}=starchyield{\left(stress\right)}_{GXEI}/(starchyield{\left(control\right)}_{GXEI})$$

RelSY = relative starch yield.

A new drought stress index DRYM (Deviation of Relative Starch Yield from Median) was then used to describe the drought tolerance because in an artificial data set this DRYM index proved superior in differentiating between drought-sensitive and drought-tolerant potato varieties independent from the yield potential^81^ compared to three other usually applied drought indices as stress susceptible index (SSI)^85^, stress tolerance index (STI) or the geometric mean productivity (GMP)^86^. The DRYM was calculated as given in Eq. (2).

2 $$DRY{M}_{GxEi}=RelS{Y}_{GxEi}-median (relS{Y}_{GxEi})$$

A positive DRYM indicates drought tolerance compared to the population median, a negative DRYM drought sensitivity. To identify the 20 most drought-tolerant (tolerant bulk) and most drought-sensitive (sensitive bulk) F1-clones, the mean DRYM was calculated for each F1 genotype and each experiment. Subsequently, genotypes were ranked (Proc Rank) by tolerance for each experiment and the mean rank was calculated. Outliers were flagged (for the method see^82^). For the remaining F1-clones, the mean DRYM and minimum, maximum and mean of the rank were calculated and the 20 F1-clones with the most extreme ranks were selected into the bulks. The mean DRYM values for the two contrasting bulks are shown as box plots. The box plot was created using ggplot2 in R version 4.1.2^87^. The significance of the differences between the bulks was calculated by applying the Welch two-sample t-test in R^88^. For SeqSNP analyses, the same ranking for drought tolerance according to the DRYM values was applied as in the previously performed association studies using the same 34 potato varieties and applying microsatellite markers (Supplementary Table S6)^27^.

### Whole-genome sequencing

The two DNA samples of the parental cultivars as well as pooled DNA from the two bulks (drought-tolerant and drought-sensitive) were sequenced on an Illumina HiSeq platform by GENEWIZ using a high output mode and a sequencing configuration of paired-end 2 × 150 bp reads within the Illumina TrueSeq Paired-End Sequencing workflow. Genome coverage of 120 × was targeted to address the genomic constitution of highly heterozygous tetraploid potatoes.

### Mapping and variant calling

The data were analysed by GENEWIZ using the Dynamic Read Analysis for GENomics (DRAGEN) platform, which is based on the DRAGEN Bio-IT Processor and optimized to handle Illumina HiSeq High data. The DRAGEN platform was used in combination with GATK (Genome Analysis Toolkit) for read mapping and identification of variants^89,90^. After sequencing, base calls and quality scores were stored in bcl format and converted into fastq files. The adapters of the reads in fasta format were trimmed with Illumina bcl2fastq v. 2.17 and low-quality sequences were identified and discarded. The published diploid potato genome assembly DM v4.03 derived from the doubled monoploid potato (*S. tuberosum* Group Phureja) clone DM1-3 516 R44^38^ was used as a reference genome for mapping and variant calling using the DRAGEN variant calling algorithm. The threshold for emitting a call was set to a minimum of 30 in variant call quality. The genomic variant annotation program SnpEff was used for the annotation and prediction of the effects of variants against the reference genome DM v6.01^91^. The files were delivered in fastq format for the sequencing results, BAM for the alignment results and VCF for the variant results.

### Rolling window analysis

To obtain a basic overview of the differences in SNP alleles, frequency plots were created, comparing the frequencies of the parent cultivars Albatros and Euroresa as well as the drought-tolerant and drought-sensitive bulk. Due to the high number of SNPs per cultivar ranging from 21.2 to 25 million, windows in which the SNP frequencies were summed had to be utilized for visualization purposes. WindowScanR was used to visualize differences in SNP frequencies^92^. The R package 4.2.0 offers the function to calculate statistics in sliding windows, either using rolling or position-based windows. For this study, rolling windows were used with the settings win_size = 1,000,000, win_step = 500,000 and funs = c(“mean”, ”sd”). The results were plotted with ggplot2^87^. In addition, CMplot in R 4.2.0 was used to obtain the SNP density graphs for Albatros and Euroresa.

### Identifying QTLs

Separation of the segregating individuals into drought-tolerant (minor yield losses under water limiting conditions) and drought-sensitive bulks was used to define regions with significant differences in SNP allele frequency as quantitative trait loci (QTLs) associated with drought tolerance. The DRYM values of the F1 population E × A had been before plotted in R with the hist() function showing an approximately normal distribution, suggesting that the trait was quantitative (Supplementary Fig. S2). CLC Genomics Workbench 21.0 was used for the genome wide comparison of SNPs between the sequences of bulks and parent cultivars with the diploid reference genome DM v4.03^38^. Afterwards, the G′-algorithm within the R package QTLseqr v0.7.5.2^93^ was applied to analyse SNP allele frequency differences. QTLseqr offers two statistical approaches for the calculation of QTLs based on SNP allele frequency differences applying NGS-BSA: QTL-seq and G′^94^. The G′-algorithm calculates normalized G values for every SNP in a tricube smoothing window depending on their distance to the focal SNP of a smoothing window. To ease the identification of QTLs and reduce noise, the SNPs from the bulks were filtered. SNPs with reads per base pair above 360 and reads per base pair below 20 were excluded. Moreover, SNPs with allele frequencies below 10% and above 90% were excluded, and a GQ value of at least 99 was required for further calculations. The filtering step reduced the starting number of SNPs from approximately 20 million to approximately 6 million SNPs to be used as input for QTLseqr. The settings used for runGprimeAnalysis were windowSize = 1e6 and filterThreshold = 0.1. The results were plotted with a Bonferroni corrected threshold of p < 0.01.

### Detailed analysis of QTLs

Scripts for the QTL analysis were written in Perl v5.32.1. This included a script to estimate sizes of QTLs, the number of genes inside a QTL and the number of SNPs in genes within a QTL. The second step of the QTL analysis was to identify possible candidate SNPs. SNPs suitable as markers for drought tolerance were identified using two different approaches. In the first round, only SNPs exclusively present in one of the two bulks for drought tolerance, either drought-tolerant or drought-sensitive, were extracted. Then, SNPs in genes described for their influence on abiotic stress response and plant drought tolerance in the literature were selected from these. Apart from the coding sequences, SNPs located in the 5′ UTR (2000 bp) and in the 3′ UTR (500 bp) were included in these analyses. In the second round, SNPs were selected by mutation type. Only SNPs resulting in missense (amino acid exchange) and nonsense (premature stop codon) mutations were selected. Candidate genes under the detected QTL identified by the SNP analyses were visualized with RIdeogram^95^. SNP distributions per QTL at the whole genome level were compared using a Perl script. The R package Vennerable was applied to visualize the data as Venn diagrams. Inputs for the comparison were unfiltered SNPs and filtered SNPs after the comparison as described previously^96^. The unfiltered SNPs were used to reduce the number of false unique SNPs per cultivar, which occurred in some cases, in which a SNP was present in both cultivars, but one SNP did not meet the filtering criteria. CLC Genomics Workbench 21.0 was used for further visualization of SNPs and genes^97^.

### SeqSNP analyses of the association panel

Further verification of SNPs relevant for drought tolerance was achieved by using SeqSNP analysis of selected SNPs for association studies in a panel of 34 potato varieties. SeqSNP analyses represents a form of targeted genotyping by sequencing performed by LGC (LGC Biosearch Technologies, United Kingdom) using specifically designed probes for next generation sequencing^98^. In total, a list of 1,034 selected SNPs in a BED format file was made available to LGC giving the exact position of the SNPs in correspondence to the potato reference genome ST4.03 (http://spuddb.uga.edu/pgsc_download.shtml, accessed on 07.01.2024). To handle the complexity and heterozygosity of the tetraploid potato genome, 200 bp up- and downstream of targeted SNPs were required as additional information for the allele-specific design of oligonucleotides prior to the targeted sequencing. One or better two oligo probes were designed for each SNP by LGC (off-targets were not allowed, annealing temperature for primers aimed at values between 45 and 60 °C). Information for the oligonucleotides derived for the SeqSNP analyses, which can be used for future selection for drought tolerance, are given in Supplementary Tables S7 and S8 (only for the significantly with drought tolerance associated SNPs). For the plant material, the plant sample collection kit provided by LGC was used. Between 7 and 9 leaf discs were punched out with a cutting tool and stored individually in a 96-well sample collection plate. Finally, a desiccant sachet was placed on top of the sealed tubes and the collection plate was shipped to LGC for genomic DNA extraction. Using the designed oligos, the surrounding areas of the SNPs were sequenced for all 34 cultivars ranked by the DRYM drought tolerance index^27^. Sequencing was performed on a NextSeq 500 v2 platform with 150 bp paired-end reads aiming at 200 × average raw coverage per sample and target. Illumina bcl2fastq v 2.17.1.14 was used for demultiplexing of all library groups, clipping of sequencing adapters and quality trimming. Reads containing Ns and above a final length of 130 bp were removed. The quality trimmed single reads were aligned against the published diploid potato genome DM v4.03 derived from the doubled monoploid potato clone DM1-3 516 R44^38^ using Bowtie2^99^, and variants were called with Freebayes v1.0.2-16^100^. Raw sequencing data, adapter clipped sequencing data and quality trimmed reads were delivered in fastq format along with FastQC reports. Alignment files were delivered in BAM format and variant call files in VCF format, as well as a spreadsheet containing all target SNPs with information about reference and alternative nucleotides for all provided samples.

### Association studies for drought tolerance

Fisher’s exact test was used to calculate significant associations. Hence, the association panel was divided into two groups according to the DRYM drought tolerance index: drought-tolerant (1t–17t) and drought-sensitive (18t–34t) cultivars (Supplementary Table S6)^27^. The association with drought tolerance was regarded as significant at p < 0.05. As a second method the Kruskal–Wallis test was used^101^. To perform the test the kruskal.test() function within the R package stats 4.3.2 was utilized. Afterwards the effect size η^2^ was calculated using the kruskal_effsize() function from the R package rstatix 0.7.2. With the release of the DM v6.1 genome assembly significant SNPs and SNPs in candidate genes were mapped to the DM v6.1 genome assembly to obtain the SNP locations also in the new assembly^102^. For the mapping process, the SNP and its 50 bp flanking sequence in each direction were extracted from the DM v4.03 genome assembly and aligned to the DM v6.1 genome assembly with BLAST^103^. The largest structural change is present on chromosome 12, which was reversed as a whole. Figures 1D–F and 3A–C use the annotation from the DM v4.03 assembly, and Fig. 4A–F use the DM v6.1 assembly annotation. To show that most SNPs in the candidate gene originate from one specific parent, the R package Gviz was used^104^. The figures are separated in tracks, starting at the top with the genome tracks, generated with the GenomeAxisTrack() function, followed by tracks showing different features generated with the AnnotationTrack() function. The different tracks were plotted with the plotTracks() function using the settings collapse = FALSE and stacking = ”dense”. Only SNPs present in either one bulk or the other are shown, whereas SNPs present in both parental varieties and bulks against the diploid potato genome were ignored. For visualization of genes, gene models were used according to the DM v6.1 genome annotation^102^.

### Plant ethic statement

IUCN guidelines were not applicable as no endangered wild species were included in the research. Experimental research and field studies complied with relevant institutional, national, and international guidelines and legislation.

### Supplementary Information

Supplementary Information 1.

Supplementary Information 2.

Supplementary Figure 1.

Supplementary Figure 2.

## Data Availability

Whole-genome sequencing data for Albatros, Euroresa and the drought-tolerant and drought-sensitive bulks are available in the NCBI Sequence Read Archive (https://www.ncbi.nlm.nih.gov/sra/) under the accession numbers SRR14400529 (HROALB, Albatros), SRR25017214 (HROEUR, Euroresa), SRR25017213 (HROEXATOL, drought-tolerant bulk), and SRR25017212 (HROEXASEN, drought-sensitive bulk).

## References

[1] Stocker, T. F. *et al.* IPCC, 2013: Climate Change 2013: The physical science basis. *Contribution of Working Group I to the Fifth Assessment Report of the Intergovernmental Panel on Climate Change. Cambridge University Press* 1535 (2013).

[2] Tardieu F, Simonneau T, Muller B (2018). The physiological basis of drought tolerance in crop plants: A scenario dependant probabilistic approach. Annu. Rev. Plant. Biol..

[3] Muthoni J, Kabira JN (2016). Potato production under drought conditions: Identification of adaptive traits. Int. J. Hortic..

[4] Food and Agriculture Organization of the United Nations. http://www.fao.org/faostat/en/#home (2022). Accessed 11 February 2022.

[5] Caliskan, M. E., Yousaf, M. F., Yavuz, C., Zia, M. A. B. & Caliskan, S. History, production, current trends, and future prospects. In Potato production worldwide (eds. Caliskan, M. E., Bakhash, A., Jabran, K.) 1–18 (Elsevier, Academic Press, 2023).

[6] Monneveux, P., Ramírez, D. & Pino, M. Drought tolerance in potato (*S. tuberosum* L.): Can we learn from drought tolerance research in cereals? *Plant Sci*. **205**, 76–86 (2013). 10.1016/j.plantsci.2013.01.011 23498865

[7] Devaux A, Goffart JP, Kromann P, Andrade-Piedra J, Polar V, Guy H (2021). The potato of the future: Opportunities and challenges in sustainable agri-food systems. Potato Res..

[8] Kirkman, M. A. Global markets for processed potato products. In *Potato Biology and Biotechnology* (eds Vreugdenhil, D. et al) 27–44 (Elsevier, 2007).

[9] McGregor, I. The fresh potato market. In *Potato Biology and Biotechnology* (eds. Vreugdenhil, D. *et al.*) 3–26 (Elsevier, 2007).

[10] Iwama, K. & Yamaguchi, J. Abiotic stresses. In: *Handbook of potato production, improvement, and postharvest management* (eds Gopal, J. & Khurana S. M.) 231–278 (Food Product Press, 2006).

[11] Hill D, Nelson D, Hammond J, Bell L (2021). Morphophysiology of potato (*Solanum tuberosum*) in response to drought stress: Paving the way forward. Front. Plant Sci..

[12] Zarzynska K, Boguszewska-Mankowska D, Nosalewicz A (2017). Difference in size and architecture of the potato cultivars root system and their tolerance to drought stress. Plant Soil Environ..

[13] Boguszewska-Mankowska D, Zarzynska K, Nosalewicz A (2020). Drought differentially affects root system size and architecture of potato cultivars with differing drought tolerance. Am. J. Potato Res..

[14] Akkamis M, Caliska S (2023). Responses of yield, quality and water use efficiency of potato grown under different drip irrigation and nitrogen levels. Sci. Rep..

[15] Djaman, K., Irmak, S., Koudahe, K. & Allen, S. Irrigation management in potato (*Solanum tuberosum* L.) production: A review. *Sustainability* **13**, 1504 (2021).

[16] Hijmans RJ (2003). The effect of climate change on global potato production. Am. J. Potato Res..

[17] Stokstad E (2019). The new potato. Science.

[18] Rykaczewska K (2017). Impact of heat and drought stresses on size and quality of the potato yield. Plant Soil Environ..

[19] Zhang S, Xu X, Sun Y, Zhang J, Li C (2018). Influence of drought hardening on the resistance physiology of potato seedlings under drought stress. J. Integr. Agr..

[20] Sprenger H (2018). Metabolite and transcript markers for the prediction of potato drought tolerance. Plant Biotechnol. J..

[21] Dahal K, Li XQ, Tai H, Creelman A, Bizimungu B (2019). Improving potato stress tolerance and tuber yield under a climate change scenario—a current overview. Front. Plant Sci..

[22] Da Ros L (2020). Drought-induced regulatory cascades and their effects on the nutritional quality of developing potato tubers. Genes.

[23] Krannich CT, Maletzki L, Kurowsky C, Horn R (2015). Network candidate genes in breeding for drought tolerant crops. Int. J. Mol. Sci..

[24] Gervais T (2021). Potato response to drought stress: Physiological and growth basis. Front. Plant Sci..

[25] Yang X (2019). Transcriptome profiling reveals effects of drought stress on gene expression in diploid potato genotype P3–198. Int. J. Mol. Sci..

[26] Chen Y (2019). Transcriptome response to drought, rehydration and re-dehydration in potato. Int. J. Mol. Sci..

[27] Schumacher, C. *et al.* Unravelling differences in candidate genes for drought tolerance in potato (*Solanum tuberosum* L.) by use of new functional microsatellite markers. *Genes* **12**, 494 (2021). 10.3390/genes12040494 PMC8067248 33800602

[28] Schumacher, C. *et al.* Genome-wide approach to identify quantitative trait loci for drought tolerance in tetraploid potato (*Solanum tuberosum* L.). *Int. J. Mol. Sci.* **22**, 6123 (2021). 10.3390/ijms22116123 PMC8201130 34200118

[29] Michelmore RW, Paran I, Kesseli RV (1991). Identification of markers linked to disease-resistance genes by bulked segregant analysis: a rapid method to detect markers in specific regions by using segregating populations. Proc. Natl. Sci. USA.

[30] Brigneti G, Garcia-Mas J, Baulcombe D (1997). Molecular mapping of the potato virus Y resistance gene *Ry*_*sto*_ in potato. Theor. Appl. Genet..

[31] Hämäläinen J (1997). Mapping and marker-assisted selection for a gene for extreme resistance to potato virus Y. Theor. Appl. Genet..

[32] van der Lee, T., Robold, A., Testa, A., van’t Klooster, J. W. & Govers, F. Mapping of avirulence genes in *Phytophthora infestans* with amplified fragment length polymorphism markers selected by bulked segregant analysis. *Genetics* **157**, 949–956 (2001). 10.1093/genetics/157.3.949 PMC1461542 11238385

[33] Strachan SM (2019). Mapping the H2 resistance effective against *Globodera pallida* pathotype Pa1 in tetraploid potato. Theor. Appl. Genet..

[34] Kaminski KP (2016). Next generation sequencing bulk segregant analysis of potato support that differential flux into the cholesterol and stigmasterol metabolite pools is important for steroidal glycoalkaloid content. Potato Res..

[35] Takagi H (2012). Genome sequencing reveals agronomically important loci in rice using MutMap. Nat. Biotechnol..

[36] Dardick C (2013). PpeTAC1 promotes the horizontal growth of branches in peach trees and is a member of a functionally conserved gene family found in diverse plants species. Plant J..

[37] Zhao G (2019). A comprehensive genome variation map of melon identifies multiple domestication events and loci influencing agronomic traits. Nat. Genet..

[38] Potato Genome Sequencing Consortium (2011). Genome sequence and analysis of the tuber crop potato. Nature.

[39] Hamilton JP (2011). Single nucleotide polymorphism discovery in elite north American potato germplasm. BMC Genomics.

[40] Felcher KJ (2012). Integration of two diploid potato linkage maps with the potato genome sequence. PLoS ONE.

[41] Díaz P, Sarmiento F, Mathew B, Ballvora A, Mosquera Vásquez T (2021). Genomic regions associated with physiological, biochemical and yield-related responses under water deficit in diploid potato at the tuber initiation stage revealed by GWAS. PLoS ONE.

[42] van Muijen D, Anithakumari AM, Maliepaard C, Visser RG, van der Linden CG (2016). Systems genetics reveals key genetic elements of drought induced gene regulation in diploid potato. Plant Cell Environ..

[43] Gazendam I, Mojapelo P, Bairu MW (2022). Potato cultivar identification in South Africa using a custom SNP panel. Plants.

[44] Jones A, Davies HM, Voelker TA (1995). Palmitoyl-acyl carrier protein (ACP) thioesterase and the evolutionary origin of plant acyl-ACP thioesterases. Plant Cell.

[45] Asakura H (2021). Transcriptomic and metabolomics analysis provide insights into the upregulation of fatty acid and phospholipid metabolism in tomato fruit under drought stress. J. Agric. Food Chem..

[46] Hou Q, Ufer G, Bartels D (2016). Lipid signalling in plant responses to abiotic stress. Plant Cell Environ..

[47] Jetter R, Kunst L (2008). Plant surface lipid biosynthetic pathways and their utility for metabolic engineering of waxes and hydrocarbon biofuels. Plant J..

[48] Bonaventure G, Salas JS, Pollard MR, Ohlrogge JB (2003). Disruption of the *FATB* gene in Arabidopsis demonstrates an essential role of saturated fatty acids in plant growth. Plant Cell.

[49] Ali B (2021). A comprehensive study of epicuticular wax biosynthesis mechanisms and the related genes. IRJMETS.

[50] Zhang L (2013). Transgenic poplar „NL895“ expressing CpFATB gene shows enhanced tolerance to drought stress. Acta. Physiol. Plant.

[51] Arefian M, Vessal S, Malekzadeh-Shafroudi S, Siddique KHM, Bagheri A (2019). Comparative proteomics and gene expression analyses revealed responsive proteins and mechanisms for salt tolerance in chickpea genotypes. BMC Plant Biol..

[52] Scherer GFE (2011). AUXIN-BINDING-PROTEIN1, the second auxin receptor: what is the significance of a two-receptor concept in plant signal transduction?. J. Exp. Bot..

[53] Hedden P (2020). The current status of research on gibberellin biosynthesis. Plant Cell Physiol..

[54] Shohat H, Eliaz NI, Weiss D (2021). Gibberellin in tomato: Metabolism, signaling and role in drought responses. Mol. Hortic..

[55] Sirohi P, Yadav BS, Afzal S, Mani A, Singh NK (2020). Identification of drought stress-responsive genes in rice (*Oryza sativa*) by meta-analysis of microarray data. J. Genet..

[56] Karimi M (2019). Plant growth retardants (PGRs) affect growth and secondary metabolite biosynthesis in *Stevia rebaudiana* Bertoni under drought stress. S. Afr. J. Bot..

[57] Stacey MG (2016). Identification of homogentisate dioxygenase as a target for vitamin E biofortification in oilseeds. Plant Physiol..

[58] Upadhyaya, D. C. *et al.* Genetic engineering of potato (*Solanum tuberosum* L.) for enhanced α-tocopherols and abiotic stress tolerance. *Physiol. Plant.* **173**, 116–128 (2021). 10.1111/ppl.13252 33099781

[59] Kim SE (2021). Overexpression of 4-hydroxyphenylpyruvate dioxygenase (IbHPPD) increases abiotic stress tolerance in transgenic sweet potato plants. Plant Physiol. Biochem..

[60] Shaar-Mosche L, Hübner S, Peleg Z (2015). Identification of conserved drought-adaptive genes using a cross-species meta-analysis approach. BMC Plant Biol..

[61] Zhang T, Li C, Li D, Yang X (2020). Roles of YABBY transcription factors in the modulation of morphogenesis, development, and phytohormone and stress responses in plants. J. Plant Res..

[62] Bowman JL (2000). The YABBY gene family and abaxial cell fate. Curr. Opin. Plant Biol..

[63] Bowman JL, Eshed Y (2000). Formation and maintenance of the shoot apical meristem. Trends Plant Sci..

[64] Stahle MI, Kuehlich J, Staron L, von Arnim AG, Golz JF (2009). YABBYs and the transcriptional corepressors LEUNIG and LEUNIG_HOMOLOG maintain leaf polarity and meristem activity in Arabidopsis. Plant Cell.

[65] Shen Y (2022). Roles of YABBY transcription factors in the regulation of leaf development and abiotic stress responses in *Camellia sinensis*. Beverage Plant Res..

[66] Yang ZE, Gong Q, Wang LL (2018). Genome-wide study of YABBY genes in upland cotton and their expression patterns under different stresses. Front. Genet..

[67] Moumeni A (2015). Transcriptional profiling of the leaves of near-isogenic rice lines with contrasting drought tolerance at the reproductive stage in response to water deficit. BMC Genomics.

[68] Yamada T (2011). Ancestral expression patterns and evolutionary diversification of *YABBY* genes in angiosperms. Plant J..

[69] Zhao SP (2017). Genome-wide analysis of the YABBY family in soybean and functional identification of *GmYABBY10* involvement in high salt and drought stresses. Plant Physiol. Biochem..

[70] Yang H (2019). Overexpression of a soybean YABBY gene, *GmFILa*, causes leaf curling in *Arabidopsis thaliana*. BMC Plant Biol..

[71] Xia, J. *et al.* Genome-wide analysis of the YABBY transcription factor family in rapeseed (*Brassica napus* L.). *Genes* **12**, 981 (2021). 10.3390/genes12070981 PMC8306101 34199012

[72] Jia H (2016). A Raf-like MAPKKK gene, *GhRaf19*, negatively regulates tolerance to drought and salt and positively regulates resistance to cold stress by modulating reactive oxygen species in cotton. Plant Sci..

[73] Soma F, Takahashi F, Suzuki T, Shinozaki K, Yamaguchi-Shinozaki K (2020). Plant Raf-like kinases regulate the mRNA population upstream of ABA-unresponsive SnRK2 kinases under drought stress. Nat. Commun..

[74] Wang B (2018). The RAF-like mitogen-activated protein kinase kinase kinases RAF22 and RAF28 are required for the regulation of embryogenesis in Arabidopsis. Plant J..

[75] Bulgakov VP, Avramenko TV (2020). Linking brassinosteroid and ABA signaling in the context of stress acclimation. Int. J. Mol. Sci..

[76] Drabkova LZ, Honys D, Motyka V (2021). Evolutionary diversification of cytokinin-specific glucosyltransferases in angiosperms and enigma of missing cis-zeatin o-glucosyltransferase gene in Brassicaceae. Sci. Rep..

[77] Wang M, Zhang H, Zhao X, Zhou J, Qin G, Liu Y, Kou X, Zhao Z, Wu T, Zhu JK, Feng X, Li L (2023). SYNTAXIN OF PLANTS81 regulates root meristem activity and stem cell niche maintenance via ROS signaling. Plant Physiol..

[78] Hundertmark M, Hincha DK (2008). LEA (Late Embryogenesis Abundant) proteins and their encoding genes in Arabidopsis thaliana. BMC Genomics.

[79] Obidiegwu J, Bryan G, Jones H, Prashar A (2015). Coping with drought: stress and adaptive responses in potato and perspectives for improvement. Front. Plant Sci..

[80] Doyle JJ, Doyle JL (1990). Isolation of plant DNA from fresh tissue. Focus.

[81] Sprenger H (2015). Assessment of drought tolerance and its potential yield penalty in potato. Funct. Plant Biol..

[82] Sprenger H (2016). The drought response of potato reference cultivars with contrasting tolerance. Plant Cell Environ..

[83] Haas M (2020). Can metabolite- and transcript-based selection for drought tolerance in *Solanum tuberosum* replace selection on yield in arid environments?. Front. Plant. Sci..

[84] Köhl, K. I. Selection and validation experiment comparing phenotypic and marker-assisted selection for drought tolerance in *Solanum tuberosum* ssp. *tuberosum*. E!DAL electronic data archive library. 10.5447/ipk/2020/18 (2018).

[85] Fischer, R.A. & Maurer, R. Drought resistance in spring wheat cultivars. I Grain yield responses. *Aust. J. Agric. Res.* **29**, 897–912 (1978).

[86] Fernandez, G.C.J. Effective selection criteria for assessing stress tolerance. In: Adaptation of food crops to temperature and water stress, Kuo C.G. ed., Asian Vegetable Research and Development Center, Shanhuan 1992, 257–270.

[87] Wickham, H. ggplot2: Elegant Graphics for Data Analysis. ISBN 978–3–319–24277–4, https://ggplot2.tidyverse.org (Springer-Verlag New York, 2016).

[88] Welch BL (1947). The generalization of `Student’s’ problem when several different population variances are involved. Biometrika.

[89] DRAGEN. DRAGEN pipeline. www.edicogenome.com/dragen_bioit_platform/. (2021).

[90] Mckenna A (2010). The Genome Analysis Toolkit: A MapReduce framework for analyzing next-generation DNA sequencing data. Genome Res..

[91] Cingolani P (2012). A program for annotating and predicting the effects of single nucleotide polymorphisms, SnpEff: SNPs in the genome of *Drosophila melanogaster* strain w1118; iso-2; iso-3. Fly.

[92] Tavares, H. WindowScanR. GitHub https://github.com/tavareshugo/WindowScanR. (2018)

[93] Mansfeld B, Grumet R (2018). QTLseqr: An R package for bulk segregant analysis with next-generation sequencing. The Plant Genome.

[94] Magwene P, Willis J, Kelly J (2011). The statistics of bulk segregant analysis using next generation sequencing. PLoS Comput. Biol..

[95] Hao Z (2020). RIdeogram: drawing SVG graphics to visualize and map genome-wide data on the idiograms. PeerJ Comput. Sci..

[96] Swinton, J. Venn and Euler area-proportional diagrams, Vennerable. GithHub https://github.com/js229/Vennerable. (2016).

[97] QIAGEN. CLC Genomics Workbench 21.0. (2021).

[98] LGC Biosearch Technologies. SeqSNP Service Guidance Notes; Available online: https://de.scribd.com/document/654669009/seqsnp-service-guidance-notes, accessed 08.01.2024

[99] Langmead B, Salzberg SL (2012). Fast gapped-read alignment with Bowtie2. Nat. Methods.

[100] Garrison, E. & Marth, G. Haplotype-based variant detection from short-read sequencing. *arXiv preprint* arXiv:1207.3907 (2012).

[101] Tomczak, M. & Tomczak, E. The need to report effect size estimates revisited. An overview of some recommended measures of effect size. *Trends Sport Sci.* **1**(21), 19–25 (2014).

[102] Pham, G. M. *et al.* Construction of a chromosome-scale long-read reference genome assembly for potato. *GigaScience* **9**, giaa100 (2020). 10.1093/gigascience/giaa100 PMC7509475 32964225

[103] Altschul SF, Gish W, Miller W, Meyers EW, Lipman DJ (1990). Basic local alignment search tool. J. Mol. Biol..

[104] Hahne, F. & Ivanek, R. Statistical genomics: Methods and protocols. In: *Visualizing genomic data using Gviz and Bioconductor* (eds. Mathé, E. & Davis, S.) 335–351 (Springer New York, 2016). 10.1007/978-1-4939-3578-9_16 27008022

