# Supplementary material for: Whole-genome sequencing of tetraploid potato varieties reveals different strategies for drought tolerance
Source: Sci Rep. 2024 Mar 5;14:5476. doi: 10.1038/s41598-024-55669-3 (PMC10914802; doi:10.1038/s41598-024-55669-3)
Supplement: Supplementary file 1 — Supplementary Information 1. [file 41598_2024_55669_MOESM1_ESM.docx]

Supplementary Data

**Supplementary Table 1:** Overview of the samples and results of the whole-genome sequencing and SNP calling in comparison to the sequenced diploid reference genome [38]. The Sample ID is a combination of the location Rostock (HRO) and the name of the cultivar Albatros (ALB or A) and Euroresa (EUR or E). The F_1_-Bulks also have SEN or TOL added for drought-sensitive or drought-tolerant, respectively.

| **Individuals per bulk** | **Sample ID** | **Total variants** | **Total SNPs** | **Total INDELs** |
| --- | --- | --- | --- | --- |
| 1 | HROALB | 21,190,336 | 18,502,085 | 2,688,251 |
| 1 | HROEUR | 25,098,177 | 19,560,602 | 2,818,128 |
| 20 | HROEXASEN | 24,272,018 | 21,394,166 | 2,877,852 |
| 20 | HROEXATOL | 24,057,945 | 21,200,308 | 2,857,637 |

**Supplementary Table 3:** Association with drought tolerance in the potato association panel and classification of candidate SNPs. Polymorphisms (SNP ID) are shown with indication of the exact Fisher test p-value, Kruskal-Wallis test p-value, effect size η², the chromosome, the position on the chromosome, the nucleotide (Ref) for the reference genome at the position of the SNP and the alternative nucleotide (Alt) for the position of the SNP. Significance levels: p<0.05 *, p <0.01**, p=<0.001***. The p-values were obtained from the SeqSNP analyses. Gene ID, position, type of mutation and gene name are given for each significantly associated SNP. SNPs were remapped to the genome assembly and annotation v6.1 for *S. tuberosum* Group Phureja DM 1-3 516 R44 [33]. Hence, locations and gene IDs are according to the v6.1 genome annotation. The corresponding position in the former v4.3 genome annotation is given in brackets.

| SNP ID | Chr | Position | Ref | Alt | p-Value (Fisher) | p-Value Kruskal-Wallis | η² | Type | Gene ID | Gene Name |
| --- | --- | --- | --- | --- | --- | --- | --- | --- | --- | --- |
| Soltu.DM.02G024960.1_SNP1 | ST4.03ch02 | 38,180,115  [40,335,918] | T | G | 0.2273 | 0.01941* | 0.14 | Synonymous Variant | Soltu.DM.02G024960.1 | *StSYP* |
| Soltu.DM.02G025020.1_SNP1 | ST4.03ch02 | 38,227,327  [40,383,288] | T | C | 0.1694 | 0.04765* | 0.0913 | Intron Variant | Soltu.DM.02G025020.1 | *StLEA* |
| Soltu.DM.06G033680.1_SNP5 | ST4.03ch06 | 57,938,560  [58,293,229] | C | G | 0.0184* | 0.04206* | 0.0979 | Missense variant | Soltu.DM.06G033680.1 | *StFATA* |
| Soltu.DM.06G034890.1_SNP2 | ST4.03ch06 | 58,819,351  [59,199,152] | T | C | 0.0167* | 0.2659 | 0.00743 | 5ʼ-UTR variant | Soltu.DM.06G034890.1 | *StABP1* |
| Soltu.DM.12G026420.1_SNP1 | ST4.03ch12 | 56,378,682  [3,034,965] | C | A | 0.0255* | 0.08694 | 0.0603 | 3ʼ-UTR variant | Soltu.DM.12G026420.1 | *StPKSP1* |
| Soltu.DM.12G026450.1_SNP1 | ST4.03ch12 | 56,390,498  [3,046,881] | T | C | 0.0134* | 0.005702** | 0.208 | 3ʼ-UTR variant | Soltu.DM.12G026450.1 | *StPKSP2* |
| Soltu.DM.12G026610.1_SNP1 | ST4.03ch12 | 56,512,063  [2,910,964] | G | C | 0.1571 | 0.01831* | 0.143 | Missense variant | Soltu.DM.12G026610.1 | *StBRI1* |
| Soltu.DM.12G026680.1_SNP2 | ST4.03ch12 | 56,600,071  [2,821,461] | C | T | 0.0004*** | 0.01019* | 0.175 | 3ʼ-UTR variant | Soltu.DM.12G026680.1 | *StYAB5* |
| Soltu.DM.12G026680.1_SNP1 | ST4.03ch12 | 56,600,575  [2,820,957] | T | G | 0.0039** | 0.002302** | 0.259 | 3ʼ-UTR variant |  |  |
| Soltu.DM.12G027240.1_SNP1 | ST4.03ch12 | 57,286,822  [2,397,900] | G | A | 0.1411 | 0.04104* | 0.0992 | Missense variant | Soltu.DM.12G027240.1 | *StZOG1* |
| Soltu.DM.12G029920.1_SNP4 | ST4.03ch12 | 59,287,788  [363,947] | C | A | 0.0149* | 0.01298* | 0.162 | Missense variant | Soltu.DM.12G029920.1 | *StHGD* |

**Supplementary Table 4**: Location of candidate genes in the DM v6.1 [102] and DM v4.03 [38].

|  | **Gene** | **ID (v6.1)** | **Chr (v6.1)** | **Pos (v6.1)** | **ID (v4.03)** | **Chr (v4.03)** | **Pos (v4.03)** |
| --- | --- | --- | --- | --- | --- | --- | --- |
| *StSYP* | Syntaxin | Soltu.DM.02G024960.1 | 2 | 38,179,246 – 38,180,145 | PGSC0003DMG400003603 | 2 | 40,335,048 – 40,335,948 |
| *StLEA* | Late embryogenesis abundant | Soltu.DM.02G025020.1 | 2 | 38,224,343 – 38,229,308 | PGSC0003DMG400003600 | 2 | 40,382,734 – 40,385,269 |
| *StFATA* | Acyl-ACP thioesterase | Soltu.DM.06G033680.1 | 6 | 57,933,541 – 57,938,992 | PGSC0003DMG400020163 | 6 | 58,288,217 – 58,294,124 |
| *StABP1* | ER auxin binding protein 1 (ABP1) | Soltu.DM.06G034890.1 | 6 | 58,822,497 – 58,818,364 | PGSC0003DMG401020044 | 6 | 59,197,495 – 59,201,044 |
| *StKS* | ent-kaurene synthase B | Soltu.DM.07G028660.1 | 7 | 57,590,958 - 57,597,303 | PGSC0003DMG400022114 | 7 | 56,710,790 – 56,715,111 |
| *StPKSP1* | Protein kinase superfamily protein | Soltu.DM.12G026420.1 | 12 | 56,378,013 – 56,378,722 | PGSC0003DMG401002925 | 12 | 3,034,851 – 3,042,007 |
| *StPKSP2* | Protein kinase superfamily protein | Soltu.DM.12G026450.1 | 12 | 56,389,931 – 56,390,647 | PGSC0003DMG402002925 | 12 | 3,046,876 – 3,055,218 |
| *StBRI1* | Serine/threonine-protein kinase BRI1 | Soltu.DM.12G026610.1 | 12 | 56,512,114 – 56,510,417 | PGSC0003DMG402002888 | 12 | 2,910,279 – 2,912,610 |
| *StYAB5* | YABBY domain class transcription factor | Soltu.DM.12G026680.1 | 12 | 56,601,086 – 56,595,140 | PGSC0003DMG400002883 | 12 | 2,820,516 – 2,827,260 |
| *StZOG1* | Zeatin O-glucosyltransferase | Soltu.DM.12G027240.1 | 12 | 57,287,465 – 57,285,879 | PGSC0003DMG400007833 | 12 | 2,397,313 – 2,398,696 |
| *StHGD* | Homogentisate 1,2-dioxygenase | Soltu.DM.12G029920.1 | 12 | 59,284,492 – 59,288,168 | PGSC0003DMG400015330 | 12 | 363,545 – 367,266 |

**Supplementary Table 5:** Overview about QTLs associated to drought tolerance in this study overlapping with previously published QTLs or markers associated with water deficiency or drought tolerance

| **QTL ID** | **Overlaps (Markers/QTL)** | **Associated Trait** | **Reference** |
| --- | --- | --- | --- |
| QTL 6 | WDp-5.13 | Water deficiency | [41] |
| QTL 6 | PGSC0003DMG400030542 | Drought tolerance | [42] |
| QTL 7 | PGSC0003DMG400020481 | Drought tolerance | [42] |
| QTL 8 | HRO_BADH_2 | Drought tolerance | [27] |
| QTL 8 | HRO_BDGEH_1B | Drought tolerance | [27] |
| QTL 8 | HRO_NDPK2_1 | Drought tolerance | [27] |
| QTL 11 | WDp-18.8 | Water deficiency | [41] |
| QTL 11 | PGSC0003DMG400012118 | drought tolerance regulator | [42] |
| QTL 13 | HRO_ETR1_1A_a_d | Drought tolerance | [27] |
| QTL 13 | HRO_JA2_1 | Drought tolerance | [27] |
| QTL 14 | HRO_EBF1_2 | Drought tolerance | [27] |
| QTL 14 | WDp-12.14 | Water deficiency | [41] |
| QTL 15 | HRO_PARGH_1C | Drought tolerance | [27] |

**Supplementary Table 6:** Selection of the parental varieties from the association panel ranked according to the DRYM values [27]

Cultivar name, breeding companies, cultivar identifier, DRYM and drought tolerance rank (1t = most drought-tolerant, 34t = most drought-sensitive) of 34 mostly starch potato cultivars.

| Cultivar | Breeder | Cultivar ID | DRYM | Rank |
| --- | --- | --- | --- | --- |
| KOLIBRI | NORIKA | 2873 | 0.13705 | 1t |
| KARLENA | NORIKA | 2871 | 0.08962 | 2t |
| PRIAMOS | SAKA | 2879 | 0.08819 | 3t |
| SOMMERGOLD | FIRLBECK | 2868 | 0.0596 | 4t |
| POWER | FIRLBECK | 2867 | 0.0574 | 5t |
| ALBATROS | NORIKA | 2870 | 0.05042 | 6t |
| SIBU | SAKA | 2881 | 0.04748 | 7t |
| VERDI | SAKA | 2882 | 0.03717 | 8t |
| SATURNA | EUROPLANT | 2675 | 0.03639 | 9t |
| ELDENA | EUROPLANT | 2854 | 0.02836 | 10t |
| DESIREE |  | 382 | 0.02191 | 11t |
| GOLF | SAKA | 2878 | 0.02017 | 12t |
| KIEBITZ | NORIKA | 2872 | 0.01824 | 13t |
| ULME | BAVARIA | 2853 | 0.01636 | 14t |
| JUMBO | FIRLBECK | 2864 | 0.01377 | 15t |
| EURONOVA | EUROPLANT | 2857 | 0.00923 | 16t |
| TOMBA | EUROPLANT | 2863 | 0.00851 | 17t |
| MAXILLA | NORIKA | 2875 | 0.00684 | 18t |
| BURANA | SAKA | 2877 | 0.00627 | 19t |
| KORMORAN | NORIKA | 2874 | 0.00216 | 20t |
| EUROSTARCH | EUROPLANT | 2859 | -0.0072 | 21t |
| ALEGRIA | NORIKA | 2673 | -0.0094 | 22t |
| TOMENSA | EUROPLANT | 2862 | -0.0131 | 23t |
| PIROL | NORIKA | 2876 | -0.02 | 24t |
| JASIA | NIEHOFF | 2869 | -0.0203 | 25t |
| LOGO | FIRLBECK | 2865 | -0.022 | 26t |
| EUROTANGO | EUROPLANT | 2860 | -0.0225 | 27t |
| MAXI | FIRLBECK | 2866 | -0.0285 | 28t |
| KURAS | EUROPLANT | 2861 | -0.035 | 29t |
| RAMSES | SAKA | 2880 | -0.036 | 30t |
| MILVA | BERDING | 2674 | -0.0412 | 31t |
| EURORESA | EUROPLANT | 2858 | -0.0425 | 32t |
| EUROBRAVO | EUROPLANT | 2855 | -0.0438 | 33t |
| EUROFLORA | EUROPLANT | 2856 | -0.055 | 34t |

**Supplementary Table 7:** Information about SNPs provided to LGC. As an example, data are shown for SNPs significantly associated with drought tolerance.

| **Chromosome** | **Start** | **End** | **SNP ID** |  |
| --- | --- | --- | --- | --- |
| ST4.03ch02 | 40,335,917 | 40,335,918 | Soltu.DM.02G024960.1_SNP1 | |
| ST4.03ch02 | 40,383,287 | 40,383,288 | Soltu.DM.02G025020.1_SNP1 | |
| ST4.03ch06 | 58,293,228 | 58,293,229 | Soltu.DM.06G033680.1_SNP5 |  |
| ST4.03ch06 | 59,199,151 | 59,199,152 | Soltu.DM.06G034890.1_SNP2 |  |
| ST4.03ch12 | 2,820,956 | 2,820,957 | Soltu.DM.12G026680.1_SNP1 |  |
| ST4.03ch12 | 2,397,899 | 2,397,900 | Soltu.DM.12G027240.1_SNP1 | |
| ST4.03ch12 | 2,821,460 | 2,821,461 | Soltu.DM.12G026680.1_SNP2 |  |
| ST4.03ch12 | 2,910,963 | 2,910,964 | Soltu.DM.12G026610.1_SNP1 | |
| ST4.03ch12 | 3,034,964 | 3,034,965 | Soltu.DM.12G026420.1_SNP1 |  |
| ST4.03ch12 | 3,046,880 | 3,046,881 | Soltu.DM.12G026450.1_SNP1 |  |
| ST4.03ch12 | 363,946 | 363,947 | Soltu.DM.12G029920.1_SNP4 |  |

**Supplementary Table 8:** Positions and oligos designed by LGC for the SeqSNP analyses (only shown for SNPs significantly associated with drought tolerance)

| **Chromosome** | **Start** | **Stop** | **Strand** | **Name** | **Sequence** | **Tm** |
| --- | --- | --- | --- | --- | --- | --- |
| ST4.03ch02 | 40,335,920 | 40,335,960 | - | Soltu.DM.02G024960.1_SNP1 | TTTCAAGTACTATCAAGAGCTCAATGTAGCAATTAAGCAG | 50.08 |
| ST4.03ch02 | 40,383,240 | 40,383,280 | + | Soltu.DM.02G025020.1_SNP1a | AATTCTGCCTAATTACGATCTAAAAAAAGAAATAAATTGA | 45.75 |
| ST4.03ch02 | 40,383,335 | 40,383,375 | - | Soltu.DM.02G025020.1_SNP1b | GGGATTAGGCCTTGGCCTTACCGCAGTTCATTGGTCCTTT | 58.00 |
| ST4.03ch06 | 58,293,172 | 58,293,212 | + | Soltu.DM.06G033680.1_SNP5 | CTCCAAAGGATGTAAACAAGAGCTTCTTGCATTTGTTGAG | 52.41 |
| ST4.03ch06 | 59,199,226 | 59,199,266 | - | Soltu.DM.06G034890.1_SNP2 | CTACAAGTTTTAGTTGTTATATCTCGTCCTCCAGTGAAAG | 49.80 |
| ST4.03ch12 | 2,397,904 | 2,397,944 | - | Soltu.DM.12G027240.1_SNP1 | CTAATTCCCTACAAGAATTCATAAACTCTCCACAATTAAG | 47.64 |
| ST4.03ch12 | 2,820,985 | 2,821,025 | - | Soltu.DM.12G026680.1_SNP1 | AGATAAATCATCAGAGAGCTTATCACTTTTATTCTTTGAC | 47.36 |
| ST4.03ch12 | 2,821,415 | 2,821,455 | + | Soltu.DM.12G026680.1_SNP2 | TCAAATAAGCAAGAACAAGGTAATTTATGTTTTGTCTCAT | 48.06 |
| ST4.03ch12 | 2,910,878 | 2,910,918 | + | Soltu.DM.12G026610.1_SNP1 | ACCATCTTGGGTGTTTAGCCTCCCTTCTTTATATATGTTG | 51.55 |
| ST4.03ch12 | 3,034,966 | 3,035,006 | - | Soltu.DM.12G026420.1_SNP1 | TAAACATTTCTCTGCTGTATTTTGTTTCTTTTCTTTAATC | 46.55 |
| ST4.03ch12 | 3,046,889 | 3,046,929 | - | Soltu.DM.12G026450.1_SNP1 | TGCATGTCAGATTTTGGCTCTGTGTTTGCTGAATTTGTTC | 54.00 |
| ST4.03ch12 | 363,980 | 364,020 | - | Soltu.DM.12G029920.1_SNP4 | TGGGCACTTGAATCTCCGTTTATGGATCACGATTATTACC | 53.25 |
