# Supplementary material for: Whole-genome sequencing of tetraploid potato varieties reveals different strategies for drought tolerance
Source: Sci Rep. 2024 Mar 5;14:5476. doi: 10.1038/s41598-024-55669-3 (PMC10914802; doi:10.1038/s41598-024-55669-3)
Supplement: Supplementary file 3 — Supplementary Figure 1. [file 41598_2024_55669_MOESM3_ESM.docx]

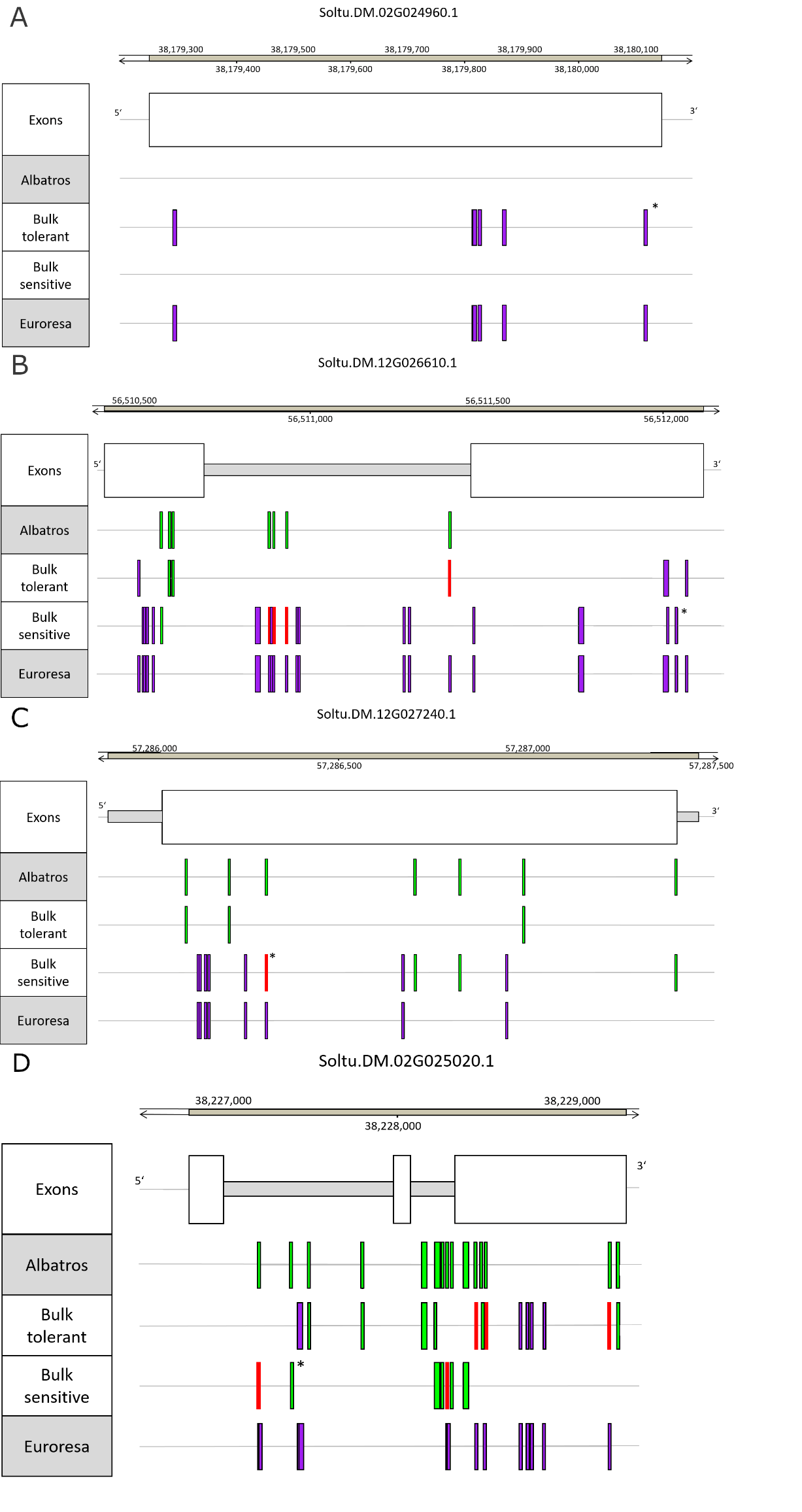


**Supplementary Figure 1.** SNP distribution in the four identified genes for drought tolerance in potato using the Kruskal-Wallis test. (**A**) Visualization of SNPs located in the gene Soltu.DM.02G024960.1 (*StSYP*) using the R package Gviz. The first track shows the location of *StSYP* (gray) according to the DM v6.1 genome annotation. The following exon track shows the exact location of the exons. The subsequent four tracks give the exact positions of SNPs, insertions and deletions in the parents Albatros and Euroresa, as well as in the drought-tolerant and drought-sensitive bulk. SNPs are shown in different colors depending on their origin: green (Albatros), purple (Euroresa) and red (present in both parents), asterisks mark significantly associated SNPs. (**B**) Visualization of SNPs located in the Soltu.DM.12G026610.1 gene (*StBRI1*) using the R package Gviz. Tracks and colors are the same as used in Fig. 4A. (**C**) Visualization of SNPs in the Soltu.DM.12G027240.1 gene (*StZOG1*) by using the R package Gviz. Tracks and colors are the same as used in Fig. 4A. (**D**) Visualization of the SNPs located in the Soltu.DM.02G025020.1 gene *(StLEA*) using the R package Gviz. Tracks and colors are the same as used in Fig. 4A.
