# Supplementary material for: Whole-genome sequencing of tetraploid potato varieties reveals different strategies for drought tolerance
Source: Sci Rep. 2024 Mar 5;14:5476. doi: 10.1038/s41598-024-55669-3 (PMC10914802; doi:10.1038/s41598-024-55669-3)
Supplement: Supplementary file 4 — Supplementary Figure 2. [file 41598_2024_55669_MOESM4_ESM.docx]

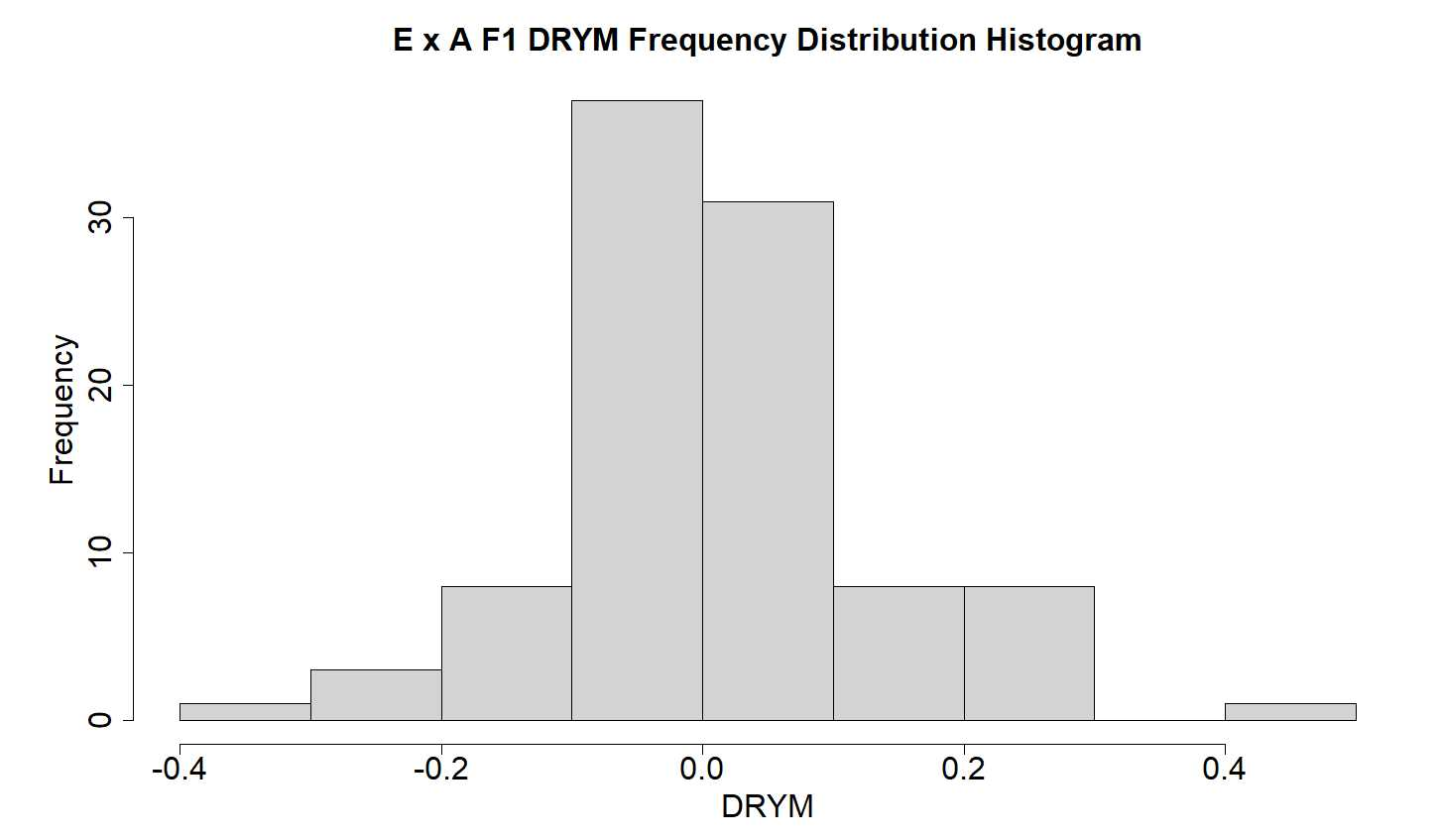


**Supplementary Figure 2:** Frequency distribution histogram showing the DRYM values for the F1 population E x A
